# Plasma-derived DNA containing-extracellular vesicles induce STING-mediated proinflammatory responses in dermatomyositis

**DOI:** 10.7150/thno.59152

**Published:** 2021-05-21

**Authors:** Yubin Li, Christina Bax, Jay Patel, Thomas Vazquez, Adarsh Ravishankar, Muhammad M. Bashir, Madison Grinnell, DeAnna Diaz, Victoria P. Werth

**Affiliations:** 1 Corporal Michael J. Crescenz Veterans Affairs Medical Center Philadelphia, PA.; 2 Department of Dermatology, School of Medicine, University of Pennsylvania, Philadelphia, PA 19104.

**Keywords:** extracellular vesicles, dsDNA, type I interferon, stimulator of interferon genes, dermatomyositis

## Abstract

**Objectives:** Extracellular vesicles (EVs) are lipid bilayer membrane vesicles that are present in various bodily fluids and have been implicated in autoimmune disease pathogenesis. Type I interferons (IFN), specifically IFN-β, are uniquely elevated in dermatomyositis (DM). The stimulator of interferon genes (STING) works as a critical nucleic acid sensor and adaptor in type I IFN signaling with possible implications in autoimmune diseases such as DM. In the current study, we investigated whether circulating EVs contribute to proinflammatory effects in DM, whether these proinflammatory responses are mediated by the STING signaling pathway, and if so, by what mechanism STING is activated.

**Methods:** We collected and characterized EVs from plasma of healthy controls (HC) and DM patients; analyzed their abilities to trigger proinflammatory cytokines release by ELISA, and explored STING signaling pathway activation using immunoblot and immunofluorescent staining. STING signaling pathway inhibitors and RNAi were used to further investigate whether STING was involved in EVs-triggered proinflammatory response. DNase/lipid destabilizing agent was utilized to digest EVs and their captured DNA contents to evaluate how EVs triggered STING-mediated proinflammatory response in DM.

**Results:** EVs isolated from DM plasma triggered proinflammatory cytokines including type I IFN release with STING signaling pathway activation. The activated STING pathway was preferentially mediated by dsDNA captured by EVs. Suppression of STING or its downstream signaling proteins attenuated the EVs-mediated proinflammatory response.

**Conclusions:** Plasma-derived, DNA containing-EVs induced STING-mediated proinflammatory effects in DM. Targeting the STING pathway may be a potential therapeutic approach for DM.

## Introduction

Dermatomyositis (DM) is a rare inflammatory autoimmune disease that mainly affects skin, muscle, and lung 1, 2. DM is difficult to diagnose and the pathogenesis of skin inflammation in DM is still not well understood 3. Previous studies have shown that type I interferon (IFN)-inducible genes are upregulated in both adult DM and juvenile DM blood samples, as well as in muscle and skin biopsies of DM patients 4, 5. Among these type I IFNs, increased IFNβ, but not IFNα or IFNκ, transcript levels were closely correlated with activation of IFN-inducible gene scores in DM skin samples 6, 7. Thus, the type I IFN signaling pathway may be important in the pathogenesis of DM 8. Nonetheless, the mechanism by which type I IFN is upregulated in DM patients is still not well understood.

Type I IFNs are primarily induced by the activation of pattern-recognition receptors, such as Toll-like receptors (TLRs), RIG-I-like receptors (RLRs), and cytoplasmic DNA sensors 9. Among cytoplasmic DNA sensors, stimulator of interferon genes (STING) works as a critical sensor and adaptor for the host immune response to cytosolic DNA and cyclic dinucleotides in type I IFN signaling 10. The STING pathway plays an important role in immunity and inflammation, and aberrant activation of this pathway is increasingly being implicated in autoimmune disease 11. Multiple stress signals, including leaked nuclear or mitochondrial DNA, viral RNA, and endoplasmic reticulum (ER) damage activate the STING pathway 12. Under normal conditions, STING is bound to the cytosolic side of ER membrane. Activated STING then activates TANK-binding kinase 1 (TBK1), a downstream protein kinase, which subsequently phosphorylates the transcription factor interferon regulatory factor 3 (IRF3) 13. This ultimately leads to increased transcription of inflammatory factors, such as interferons 13, 14. The production of NF-κB-dependent inflammatory cytokines is also observed downstream of STING activation, but the underlying mechanisms remain opaque 15.

Extracellular vesicles (EVs) are lipid bilayer membrane vesicles that exist in various bodily fluids. EVs are released by normal, diseased, and transformed cells *in vitro* and *in vivo* and are capable of carrying lipids, proteins, mRNAs, non-coding RNAs, and even DNA 16. They are abundant in serum and plasma and have been a source of considerable interest as potential disease biomarkers 17. Normally, they maintain physiological functions by transferring biological information to neighboring cells and facilitating intercellular communication, but are also involved in the pathogenesis of numerous autoimmune diseases including rheumatoid arthritis (RA), systemic lupus erythematosus (SLE), sjogren's syndrome, systemic sclerosis, and antiphospholipid syndrome 18. In DM, plasma exosomes from children with active, untreated juvenile DM are taken up by aortic endothelial cells and are associated with alterations in gene expression in those cells 19. Serum concentrations of immune cell-derived microparticles in polymyositis/dermatomyositis are also elevated 20. However, the specific role that circulating EVs may play in DM pathogenesis and their role in activation of inflammatory pathways are still not clear.

Both STING pathway activation and EVs have been implicated in autoimmune disease pathogenesis. In the current study, we investigated whether circulating EVs contribute to proinflammatory effects in DM, whether these proinflammatory responses are mediated by the STING signaling pathway, and if so, by what mechanism STING is activated.

## Materials and Methods

### Ethics Statement

The University of Pennsylvania Institutional Review Board has approved human subject involvement in this study. All subjects in the study signed an Informed Consent document before enrollment.

### Reagents and Antibodies

2'3'-cGAMP (sodium salt), STING antagonist H-151, TBK1 inhibitors Amlexanox and MRT67307 were obtained from Cayman Chemical Company (Ann Arbor, MI). Phospho-STING (Ser366) (D7C3S) rabbit antibody, Phospho-STING (Ser366) (D8K6H) rabbit antibody, Phospho-STING (Ser365) (D8F4W) rabbit antibody, STING (D2P2F) rabbit antibody, phospho-TBK1/NAK (Ser 172) (D52C2) rabbit antibody, TBK1/NAK (D1B4) rabbit antibody, phospho-IRF-3 (Ser396) (D601M) rabbit antibody, IRF-3 (D83B9) rabbit antibody, and β-Actin (8H10D10) mouse antibody were obtained from Cell Signaling Technology Company (Danvers, MA). Anti-NF-κB p65 (phosphor S536) antibody was purchased from Abcam (Cambridge, MA). CD63 rabbit antibody (25682-1-AP), CD81 mouse antibody (66866-1-Ig), and CD9 mouse antibody (60232-1-Ig) were purchased from Proteintech (Rosemont, IL). HRP conjugated goat anti-rabbit or mouse secondary antibody were obtained from Jackson ImmunoResearch Laboratories (West Grove, PA). Alexa Fluor 568 goat anti-mouse IgG and Alexa Fluor 568 goat anti-rabbit IgG secondary antibodies were obtained from Thermo Fisher (Eugene, OR).

### Peripheral blood mononuclear cells (PBMCs)

Peripheral blood mononuclear cells (PBMCs) were isolated using Ficoll-Paque gradient (GE Healthcare, Chicago, IL) and resuspended in RPMI-1640 medium with 0.2% BSA 21. PBMCs were stimulated with EVs in the presence/absence of antagonists/inhibitors for 15 h, and then supernatants and cells were collected for subsequent experiments.

### Mouse macrophages experiments

C57BL/6J (Stock No.: 000664) and C57BL/6J-Sting1gt/J (Stock No.: 017537) mice purchased from the Jackson laboratory were housed in a pathogen-free environment and given food and water *ad libitum* 22. All the animal experiments were approved by the Institutional Animal Care and Use Committee of Philadelphia VA Medical Center. Mouse peritoneal macrophage isolation was conducted with 3% thioglycolate broth (TGB) (Sigma-Aldrich, St. Louis, MO) medium induction and then isolated followed by the published protocol accordingly 23. Cells were maintained in RPMI-1640 medium with 10% heat-inactivated fetal bovine serum (FBS), 2 mM L-glutamine, 100 U/mL of penicillin, and 100 µg/mL of streptomycin.

### Isolation and purification of extracellular vesicles

Venous heparinized blood from DM patients or HCs was centrifuged at 500 g for 10 min to obtain cell-free plasma. Then the cell-free plasma was centrifuged at 2,000 g for 20 min to remove the debris. 5 mL of the obtained plasma was subsequently centrifuged at 20,000 g for 30 min, the pelleted large extracellular vesicles (lEVs) were washed and resuspended in PBS. The collected lEV-depleted supernatants were then centrifuged at 100,000 g for 2 h at 4 °C to pellet small extracellular vesicles (sEVs). The pelleted sEVs were washed in 5 mL of PBS and then centrifuged at 100,000 g for 2 h at 4 °C to purify the sEVs. Collected EVs were resuspended in 500 μL of PBS 24. sEVs were also isolated by using total exosome isolation kit (Invitrogen, CA), and 1mL of the obtained plasma-derived sEVs were resuspended in 100 µL of PBS 25.

### Enzyme pretreatment of EVs

sEVs were pretreated with/without DNase I (Thermo scientific, Waltham, MA) or dsDNase (Thermo scientific, Waltham, MA) in the presence/absence of 0.075% Triton X-100 in the 37 °C incubator for 1 h 26. The total volume of all the different pretreated sEVs was kept the same by adding PBS. After pretreatment, the sEVs were used for cell stimulation, PCR, or size distribution/concentration testing.

### Determination of concentration and size distribution of EVs

sEVs or lEVs isolated from HC and DM patients' plasma were diluted with filtered 1× PBS with 1% BSA, and analyzed through nCS1 by Nanoparticle Analyzer (NPA) technology (Spectradyne, Torrance, CA). The data were processed and exported through nCS1 by the NPA.

### Determination of surface markers of EVs

ExoView chip array with antibodies against CD81, CD63, and CD9 was performed to test surface markers of EVs 27. Briefly, HC or DM plasma were diluted in PBS to obtain EV concentration within the dynamic range of the ExoView chip assay (Boston, MA). After dilution 35 µL of samples were incubated on the ExoView Chip placed in a sealed 24 well plate for 16 hours at room temperature. The ExoView chips were then washed with PBST for 3 mins followed by 3 washes in PBS. The chips were rinsed in filtered DI water and dried. The chips were then imaged and analyzed with the ExoView R100 reader.

### Cell viability and cytotoxicity assay

Cell viability and cytotoxicity assay was detected by using Cell Counting Kit-8 (WST-8) (Abcam, Cambridge, MA). Briefly, PBMCs were placed in 96-well plates at a density of 1.5×10^6^ cells/mL, 100 µL/well. After cells were stimulated with EVs for 15 h, the plates were incubated with WST-8 in the incubator with 95% air and 5% CO_2_ at 37 °C for 1 h, and then were measured at 460 nm absorbance wavelength 28.

### Binding and uptake of EVs by PBMCs

The detection of binding and uptake of EVs by PBMCs was referenced from published literature with modification 29. Specifically, 250 μL of purified EVs were stained with the lipophilic cell tracers of CM-Dil at 2 μM concentration by incubating for 30 min at 37 °C and then resuspended in 10 mL PBS and centrifuged at 100,000g for 1h twice. 2 μM of CM-Dil dye incubated in the absence of EVs for 30 min at 37 °C and then resuspended in 10 mL PBS and centrifuged at 100,000 g twice for 1h each time were used as negative control. The labelled EVs were resuspended in 250 μL of endotoxin-free PBS, and incubated with 1.5 × 10^6^ /mL PBMCs at the 1:40 EVs:Cells ratio (volume:volume). After PBMCs were incubated with CM-Dil labelled EVs or negative control for 15 h, the cells were fixed, incubated with 4',6-diamidino-2-phenylindole (DAPI) (1 μg/mL) in PBS for 1 min and washed 3 times, and then studied by using confocal microscopy (Olympus, Japan).

### Assessment of genomic/mitochondrial DNA by PCR

Genomic/mitochondrial DNA was assessed using PCR. PCR was performed using specified primers sequences: *hCOX* forward: 5'-TTCGGCGCATGAGCTGGAGTCC-3'; *hCOX* reverse: 5'-TATGCGGGGAAACGCCATATCG-3'; *HVRII* forward: 5'-CTCACGGGAGCTCTCCATGC-3'; *HVRII* reverse: 5'-CTGTTAAAAGTGCATACCGCCA-3'; *hLMNBI* forward: 5'-AAGCAGCTGGAGTGGTTGTT-3'; *hLMNBI* reverse: 5'-TTGGATGCTCTTGGGGTTC-3'.

### Western blot analysis

Cells were harvested and suspended in radioimmunoprecipitation assay buffer (RIPA) lysis buffer (Santa Cruz Biotechnology, Dallas, TX). The lysed cells were sonicated for 15 min and then centrifuged at 12,000 g for 5 min at 4 °C. Protein concentration was determined by the bicinchoninic acid (BCA) method. Equivalent amounts of protein were loaded onto gels and separated by sodium dodecyl sulfate-polyacrylamide gel eletrophoresis (SDS-PAGE), then electrotransferred to polyvinylidene fluoride (PVDF) membranes (BioRad, Hercules, CA). The membranes were blocked in PBST containing 5% non-fat milk, and reacted with different antibodies at 4 °C overnight. After incubation with the secondary antibody conjugated with horseradish peroxidase membranes were visualized using an enhanced chemiluminescent detection kit (MilliporeSigma, Burlington, MA) 30.

### siRNA transfection

PBMCs were seeded in 12-well plates at 1 × 10^6^ cells/mL, and then transfected with 50 nM siRNA using X-tremeGENE siRNA Transfection Reagent (Roche Diagnostics, Germany). 36 h post-transfected cells were treated with EVs for 15 h as indicated, and the relevant assays were performed 31. The control siRNA and TMEM173 (STING) siRNA used were synthesized by Santa Cruz Biotechnology (Dallas, TX). TMEM173 (STING) siRNA were also labelled by siRNA labeling kit with FAM dye (Life technologies, Carlsbad, CA) according to the user guide. After the labelled siRNA was used to transfect PBMCs, the cells were fixed for immunofluorescent staining and studied by using fluorescent microscopy.

### Immunofluorescent staining and imaging

PBMCs were fixed with 4% paraformaldehyde (PFA) for 10 min and permeabilized with 0.1% Triton X-100 for 5min. The cells were then blocked with 1% BSA/PBS for 45 min and incubated with a primary antibody for 2 h and the corresponding secondary antibody for 1 h. Washes were done 3 times with PBS after each step. When desired, the cells were incubated with 4',6-diamidino-2-phenylindole (DAPI) (1 μg/mL) in PBS for 10 min and washed 3 times. Last, the cells were photographed with a Nikon Eclipse Ti microscopy. Five fields were randomly chosen for images 32.

### The enzyme-linked immunosorbent assay (ELISA)

Enzyme-linked immunosorbent assay (ELISA) was performed to measure proinflammatory cytokines IFNβ, ΤΝFα, and IL-6 levels by Human DuoSet ELISA kit (R&D systems, Minneapolis, MN) following manufacturer's instructions. High sensitivity human IFNβ ELISA Kit from PBL Assay Science (Piscataway, NJ) was also used in the studies. Briefly, PBMCs were stimulated with EVs in the presence/absence of antagonists/inhibitors for 15 h, and then supernatant was collected for ELISA. Mouse IFNβ was measured with a Mouse IFN-beta DuoSet ELISA kit (R&D systems, Minneapolis, MN) following the manufacturer's instructions. Briefly, peritoneal macrophages eluted from C57BL/6J (Stock No.: 000664) and C57BL/6J-*Sting1^gt^*/J (Stock No.: 017537) mice were stimulated with DM plasma-derived sEVs for 15 h, and then the supernatant was collected for ELISA.

### Statistical analysis

GraphPad Prism 6 (San Diego, CA) was used for all the statistical analysis. All data were presented as mean ± standard deviations (SD) unless otherwise specified. Comparison between two groups was analyzed by the Student t test. Comparisons between the groups were analyzed by one-way ANOVA and made by Student-Newman-Keuls post-hoc method. Statistical significance was set at a level of *p*<0.05.

## Results

### DM patients' plasma-derived EVs were different from that of HC

**Figure 1:**
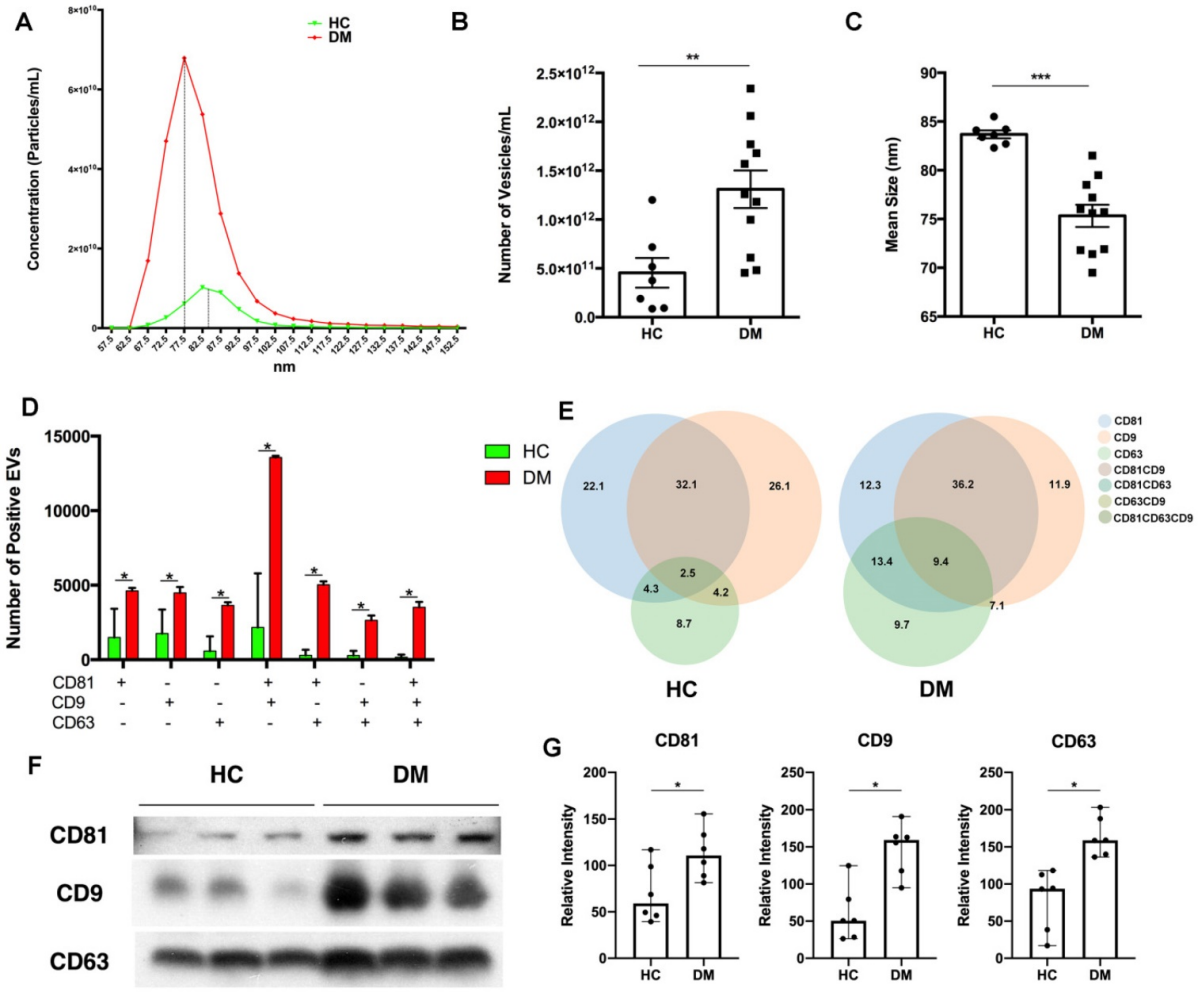
** DM plasma-derived small extracellular vesicles are different compared to HC plasma-derived small extracellular vesicles.** (A) Concentration and size distribution of small extracellular vesicles (sEVs) derived from HC plasma vs. DM plasma. (B) Total number of sEVs derived from HC plasma (4.545x10^11^ ± 4.019x10^11^ particles/mL, n = 7) and DM plasma (1.309x10^12^ ± 6.377x10^11^ particles/mL, n = 11). (C) Average size of sEVs derived from healthy plasma (83.69 ± 1.057 nm, n = 7) and DM plasma (75.33 ± 3.786 nm, n = 11). (D) Number of EVs with different surface markers CD81, CD9, and CD63 from 35 µL of HC plasma (n = 3) and DM plasma (n = 3). (E) Corresponding density of EVs with different surface makers CD81, CD9, and CD63 from HC plasma and DM plasma. (F) Representative immunoblot showing expression level of surface makers CD81, CD9, and CD63 in 0.5 µL of HC plasma (n = 3) vs DM plasma (n = 3). (G) Expression level relative intensity of CD81, CD9, and CD63 in 0.5 µL of HC plasma (n = 6) vs DM plasma (n = 6). Data in (B,C,D,G) represent mean ± SD. *P < 0.05, **P < 0.01, ***P < 0.001 between groups as indicated. Comparison between two groups was analyzed by the Student* t* test.

To explore the difference between EVs derived from DM and HC plasma, we first examined the EV concentration and size distribution. Concentrations and size distributions of EVs were analyzed through nCS1 by Spectradyne. DM plasma contained a higher sEV concentration than HC plasma (1.31×10^12^/mL vs 4.54×10^11^/mL) (**Figure 1A-B**). Although sEVs derived from HC plasma and DM plasma had a similar size distribution range (62.5 nm-142.5 nm), the mean size of HC plasma-derived sEVs was larger than that of DM patients' plasma-derived sEVs (83.69 ± 1.057 nm vs 75.33 ± 3.786 nm) (**Figure 1C**). lEVs derived from the plasma of DM patients and HC were also compared. Distinct from sEVs, the concentration of DM plasma-derived lEVs was similar to HC plasma-derived lEVs (**Figure S1A**). The mean size of DM plasma-derived lEVs had no significant difference with lEVs derived from HC plasma (**Figure S1B**).

The difference in surface markers of EVs between HC and DM plasma was also investigated. CD81, CD63, and CD9 are tetraspanin superfamily glycoproteins that are classically used as markers of exosomes, a type of sEVs. Here, CD81, CD63, and CD9 on the surface of individual EV were detected by using ExoView. DM plasma contained greater amounts of all types of EVs particles (single surface marker-positive, double surface marker-positive, and triple surface marker-positive) than HC (**Figure 1D**), which was consistent with results obtained with the nCS1 by Spectradyne (**Figure 1B**). Based on the percentage of surface markers-positive EVs, DM plasma had more EV with complex surface markers than that of healthy donors (**Figure 1E**).

Immunoblot analysis of CD81, CD63, and CD9 expression was used to further determine surface markers of EVs in the same volume of HC and DM patients' plasma. As shown in **Figure 1F**, when compared with healthy donors' plasma, DM patients' plasma exhibited higher expression of all tested EV surface markers (**Figure 1G**). However, there was no significant difference in the expression of surface markers on the same number of sEVs between HC and DM patients (**Figure S2**).

Thus, our results found that DM patients' plasma had more EVs with a smaller size than HC plasma, and that DM patients' plasma derived EVs were different from that of healthy donors.

### DM patients' plasma-derived EVs triggered proinflammatory cytokines release with STING phosphorylation

**Figure 2:**
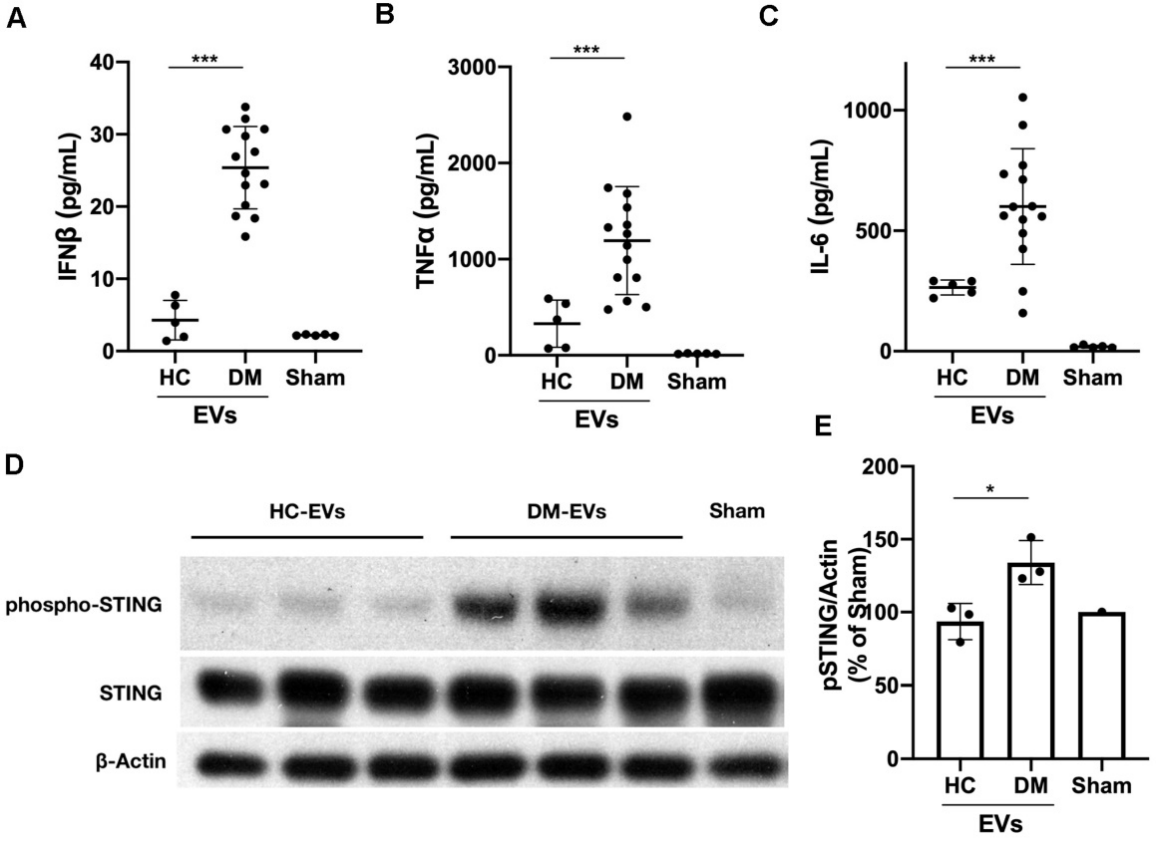
** DM plasma-derived extracellular vesicles triggered more pro-inflammatory cytokine release in PBMCs than HC plasma-derived extracellular vesicles.** 100 µL of PBS-resuspended EVs derived from 1mL plasma of 5 healthy donors and 14 DM patients were used to stimulate PBMCs (1.5x10^6^/mL, 2 mL/well) for 15 h. The supernatants were collected for interferon β (IFNβ), tumor necrosis factor α (TNFα), and interleukin 6 (IL-6) detection. (A) DM plasma-derived EVs triggered more IFNβ release (25.39 ± 5.700 pg/mL, n = 14) than HC plasma-derived EVs (4.285 ± 2.727 pg/mL, n = 5). (B) DM plasma-derived EVs triggered more TNFα release (1193 ± 561.8 pg/mL, n = 14) than HC plasma-derived EVs (329.0 ± 244.9 pg/mL, n = 5). (C) DM plasma-derived EVs triggered more IL6 release (600.2 ± 240.0 pg/mL, n = 14) than HC plasma-derived EVs (264.6 ± 31.30 pg/mL, n = 5). (D) DM plasma-derived EVs induced more STING phosphorylation than healthy plasma-derived EVs in PBMCs. (E) Relative intensity of phosphorylated STING in DM plasma- or HC plasma-derived EVs stimulated PBMCs (n = 3). Data in (A,B,C,E) represent mean ± SD. *P < 0.05, ***P < 0.001 between groups as indicated. Comparison between two groups was analyzed by the Student* t* test.

**Figure 3:**
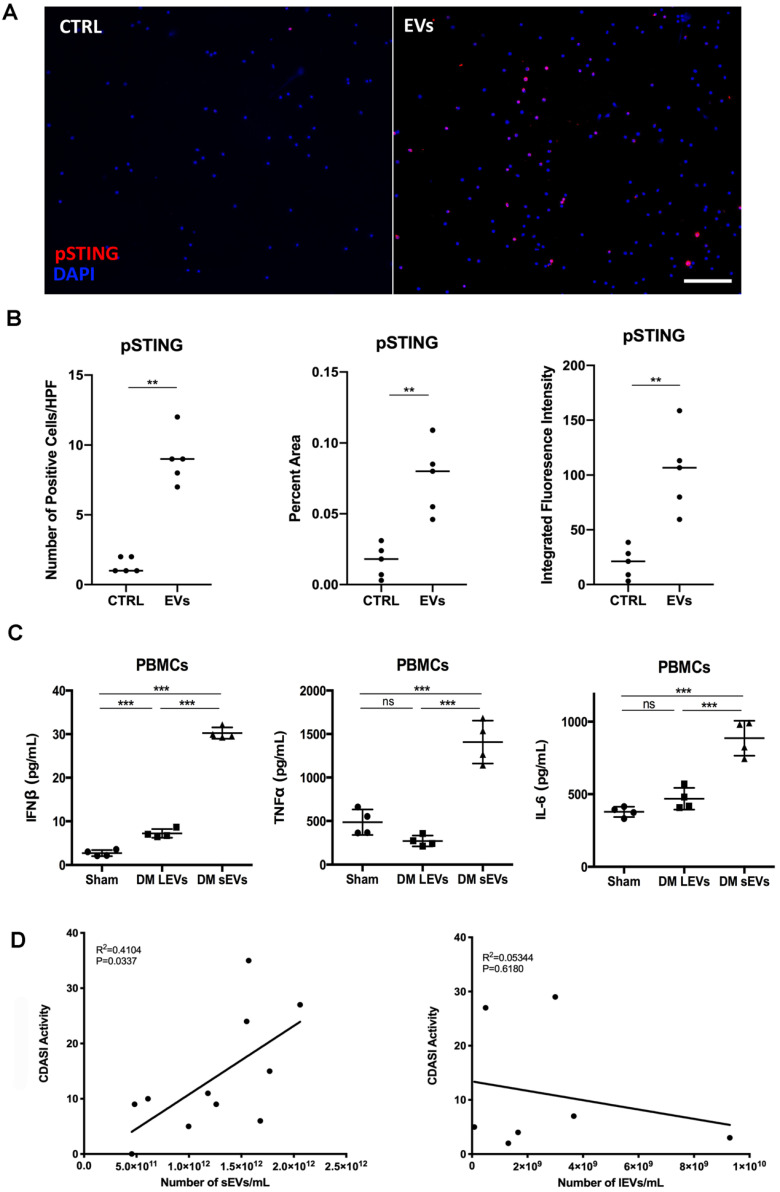
** DM plasma derived extracellular vesicles induced STING phosphorylation during their triggered pro-inflammatory response in PBMCs.** (A) Representative immunofluorescent staining images showing that EVs derived from DM plasma induced phosphorylation of STING in PBMCs. (Scale bar 100 µm) (B) Bar graph depicting the relative intensity of phosphorylated STING immunofluorescent staining in PBMCs with/without DM plasma-derived EVs stimulation (n = 5). (C) DM plasma-derived small EVs (sEVs) triggered more IFNβ release(30.24 ± 1.302 pg/mL, n = 4) than large EVs (lEVs) (7.22 ± 0.9899 pg/mL, n = 4) in PBMCs; DM plasma-derived sEVs (1407 ± 247.0 pg/mL, n = 4) triggered more TNFα release than lEVs (269.9 ± 62.53 pg/mL, n = 4) in PBMCs; DM plasma-derived sEVs (886.2 ± 120.4 pg/mL, n = 4) triggered more IL6 release than lEVs (468.7 ± 75.15 pg/mL, n = 4) in PBMCs. (D) Graph of DM Activity of Cutaneous Dermatomyositis Disease Area and Severity Index (CDASI) vs. DM sEVs concentration (n = 11) and lEVs concentration (n = 7). R^2^ = coefficient of correlation. Data in B represent median, data in C represent mean ± SD. **P < 0.01, ***P < 0.001 between groups as indicated. Comparison between two groups was analyzed by the Student* t* test.

Next, we determined whether DM and HC plasma-derived EVs could induce a proinflammatory response in circulating immune cells. 100 µL of EVs collected from 1 mL of plasma from 5 healthy donors and 14 DM patients were used to stimulate 2 mL of PBMCs for 15 hours. ELISA results showed that DM plasma-derived EVs stimulated more IFNβ, TNFα, and IL-6 release than HC plasma-derived EVs (**Figure 2A-C**). EVs were directly subjected to ELISA assay to further determine that indeed cytokines were released by EVs-stimulated PBMCs, but not secondarily EVs themselves-captured cytokines (**Figure S3**). The cell viability experiments also showed that either large EVs or small EVs derived from HC or DM plasma had no significant effects on cell viability of PBMCs when compared with non-treated PBMCs (**Figure S4**). After establishing that plasma-derived EVs in DM patients stimulate inflammatory cytokine release in PBMCs, we investigated the mechanism by which DM EVs have this effect. Lipophilic CM-DiI dye labelled DM EVs were incubated with healthy PBMCs for 15 h and then analyzed by confocal microscopy. Fluorescent images showed that EVs could be properly internalized by PBMCs, and this internalization was EV specific as PBMCs incubated with ultra-centrifuged CM-DiI dye pellet had no CM-DiI dye binding signal (**Figure S5**). Immunoblot results showed that DM patients' plasma-derived EVs induce more STING phosphorylation in PBMCs than HC EVs (**Figure 2D-E**). By using immunofluorescent staining, we also confirmed that the DM EVs-stimulated PBMCs had more phospho-STING positive fluorescence (**Figure 3A-B**).

Equal volume of lEVs and sEVs collected from 1mL of plasma were further used to investigate whether lEVs or sEVs contribute more to proinflammatory effects. Both lEVs and sEVs trigger IFNβ release in PBMCs, while sEVs induce much more IFNβ production than lEVs (**Figure 3C**). LEVs stimulation had no effects on TNFα and IL-6 production, but sEVs significantly increased TNFα and IL-6 release in PBMCs (**Figure 3C**). Thus, our results suggested that sEVs derived from DM plasma contribute more to proinflammatory cytokine release in PBMCs. Moreover, the number of sEVs, but not of lEVs, correlated with higher activity of Cutaneous Dermatomyositis Disease Area and Severity Index (CDASI) activity score, a reliable, validated outcome measure to quantify disease severity in DM patients 33 (**Figure 3D**). The demographic information including age, sex, sEVs concentration, CDASI activity score, and treatments are shown in **Table S1**.

Taken together, our findings confirmed that an equal volume of DM patients' plasma-derived EVs triggered more proinflammatory cytokine production with more phosphorylated STING than HCs' plasma-derived EVs. DM plasma-derived sEVs contributed more to proinflammatory cytokine release from PBMCs and correlated with DM disease severity.

### Inhibition of STING activation decreased DM EVs immunostimulatory effects

After finding that DM EVs increased phosphorylation of STING and increased proinflammatory cytokine release, STING antagonist H-151, which has previously demonstrated efficacy by decreasing STING phosphorylation during its inhibiting STING activation, was used to explore whether STING inhibition would block the immunostimulatory effect of DM EVs 34, 35.

**Figure 4:**
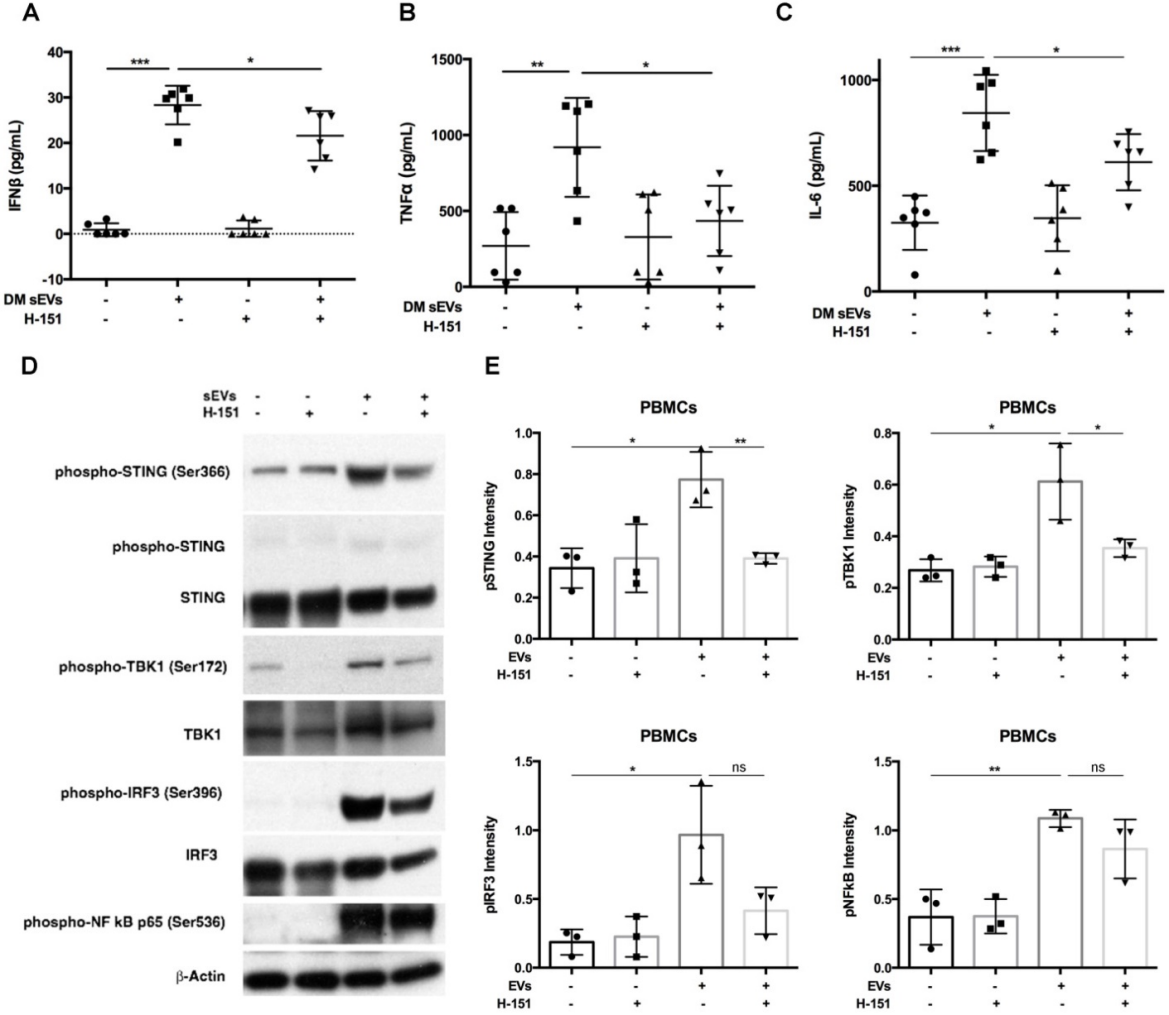
** Inhibition of STING impaired immunostimulatory effects of DM plasma derived small extracellular vesicles in PBMCs.** (A) sEVs-stimulated PBMCs secreted less IFNβ release when STING antagonist H-151 (1μM) was present ((21.58 ± 5.45 vs. 28.34 ± 4.25) pg/mL; n = 6). (B) sEVs-stimulated PBMCs secreted less TNFα release when STING antagonist H-151 was present (434.8 ± 231.5 vs. 919.1 ± 325.7) pg/mL; n = 6). (C) sEVs-stimulated PBMCs secreted less IL6 release when STING antagonist H-151 was present ((611.5 ± 132.8 vs. 844.2 ± 180.3) pg/mL; n = 6). (D) STING antagonist H-151 suppressed DM plasma-derived sEV-induced STING phosphorylation and its downstream signaling pathway TBK1, IRF3, and NFκB phosphorylation in PBMCs. (E) Relative intensity of phosphorylated STING, phosphorylated TBK1, phosphorylated IRF3, and phosphorylated NFκB in PBMCs stimulated with/without DM derived sEVs in the presence/absence of STING antagonist H-151 (n = 3). Data in (A,B,C,E) represent mean ± SD. *P < 0.05, **P < 0.01, ***P < 0.001 between groups as indicated. Comparison among three or more groups was performed using ANOVA, followed by Student-Newman-Keuls test. Comparison between two groups was analyzed by the Student* t* test.

Pre-treatment with H-151 suppressed the immunostimulatory effects of DM patients' plasma derived sEVs, significantly lowering IFNβ, TNFα, and IL-6 cytokine release (**Figure 4A-C**). Immunoblot results also showed that H-151 pretreatment inhibited STING phosphorylation by sEVs, as well as phosphorylation of its downstream signaling pathways TBK1, IRF3, and NFκB (**Figure 4D**). Quantitative analysis confirmed that sEVs derived from DM patients' plasma increased phosphorylation of STING and downstream TBK1, IRF3, and NFκB. H-151 significantly decreased STING and TBK1 phosphorylation by sEVs, and trended to suppress IRF3 and NFκB phosphorylation (**Figure 4E**). The STING agonist 2'3'-cGAMP was used as a positive control. Both 10 µg/mL and 20 µg/mL of 2'3'-cGAMP could trigger STING and its downstream TBK1 and IRF3 phosphorylation, and 1 µM of H-151 pretreatment could suppress 2'3'-cGAMP-triggered STING signaling pathway phosphorylation (**Figure S6A**). Furthermore, H-151 could also decrease 2'3'-cGAMP-induced proinflammatory cytokines release from PBMCs (**Figure S6B-D**).

Interestingly, STING antagonist H-151 also inhibited lEVs triggered IFNβ production (**Figure S7A**). However, the amount of induction of IFNβ production was less with lEVs than with sEVs. As lEVs did not trigger TNFα and IL-6 production, H-151 could not inhibit these two cytokines production (**Figure S7B-C**). LEVs also induced STING phosphorylation, and H-151 reduced lEVs-induced STING phosphorylation (**Figure S7D**).

**Figure 5:**
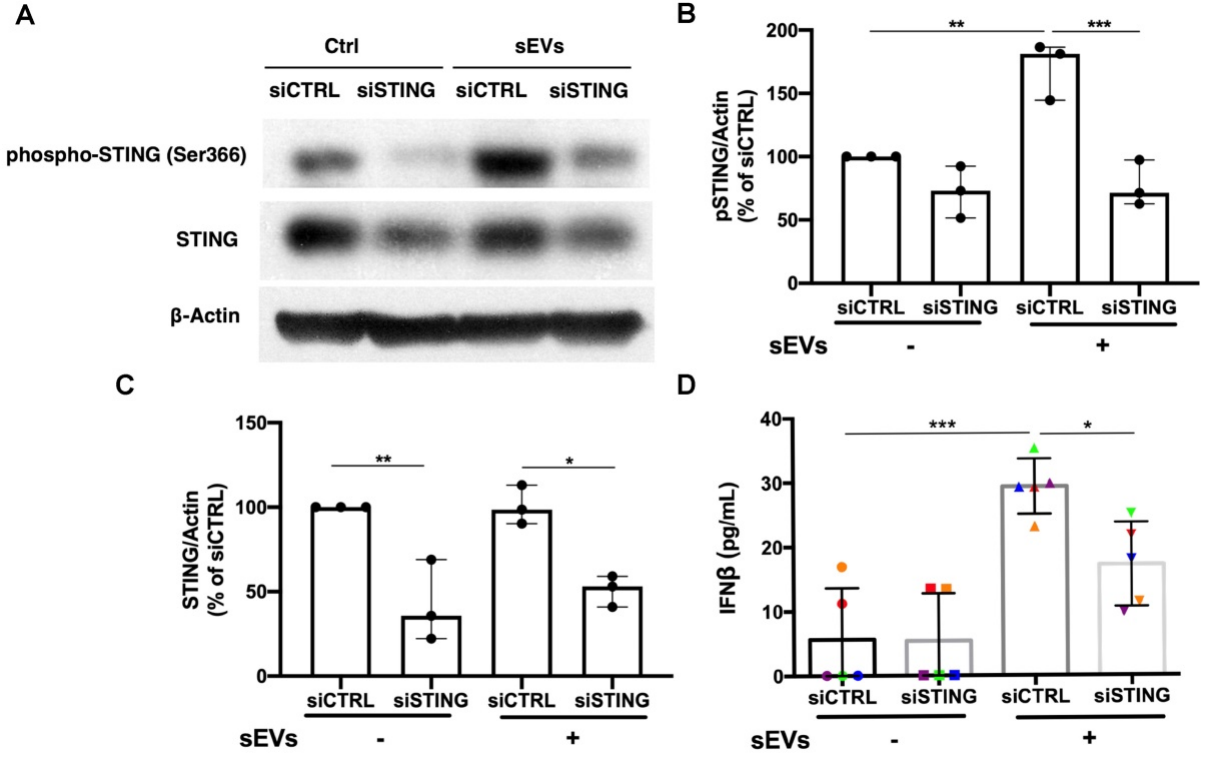
** Silencing of STING suppressed immunostimulatory effects of DM plasma derived small extracellular vesicles in PBMCs.** (A) Representative immunoblot images showed that total STING level was decreased in siSTING transfected PBMCs, and DM plasma-derived sEVs induced more STING phosphorylation in siCTRL transfected PBMCs than siSTING transfected PBMCs. (B) DM plasma-derived sEVs induced more STING phosphorylation in siCTRL transfected PBMCs than siSTING transfected PBMCs (n = 3). (C) Total STING level was decreased in siSTING transfected PBMCs (n = 3). (D) DM plasma-derived sEVs induced more IFNβ release in siCTRL transfected PBMCs (29.32 ± 4.299 pg/mL; n = 5) than siSTING transfected PBMCs (17.18 ± 6.531 pg/mL; n = 5). Data in (B,C,D) represent mean ± SD. *P < 0.05, **P < 0.01, ***P < 0.001 between groups as indicated. Comparison among three or more groups was performed using ANOVA, followed by Student-Newman-Keuls test.

Based on the STING antagonist results, we used siRNA transfection to knockdown STING to confirm the role of STING in the EVs-mediated proinflammatory response. By using immunoblot, we found that siSTING could not only reduce STING expression in PBMCs, but also suppress sEVs-mediated STING phosphorylation (**Figure 5A-C**). Cytokine ELISA results showed that when compared with siCTRL transfected PBMCs, sEVs stimulated siSTING transfected PBMCs produced less IFNβ (**Figure 5D**). Immunofluorescent staining results also showed that when compared with untreated control, FAM-labelled STING siRNA could be efficiently transfected into cells and decreased STING expression (**Figure S8**).

Peritoneal macrophages derived from C57BL/6J (Stock No.: 000664) and C57BL/6J-*Sting1^gt^*/J (Stock No.: 017537) mice were further used to explore the role of STING activation in DM plasma-derived sEVs mediated proinflammatory response. Peritoneal macrophages from C57BL/6J-*Sting1^gt^*/J had no STING expression (**Figure S9A**). DM plasma-derived sEVs could significantly trigger STING phosphorylation with IFNβ production in macrophages-derived from C57BL/6J mice but not those of C57BL/6J-*Sting1^gt^*/J mice (**Figure S9B-D**).

Taken together, these data suggested that inhibiting the STING pathway could impair the immunostimulatory effects of DM EVs.

### Inhibition of TBK1 decreased DM EVs immunostimulatory effects

**Figure 6:**
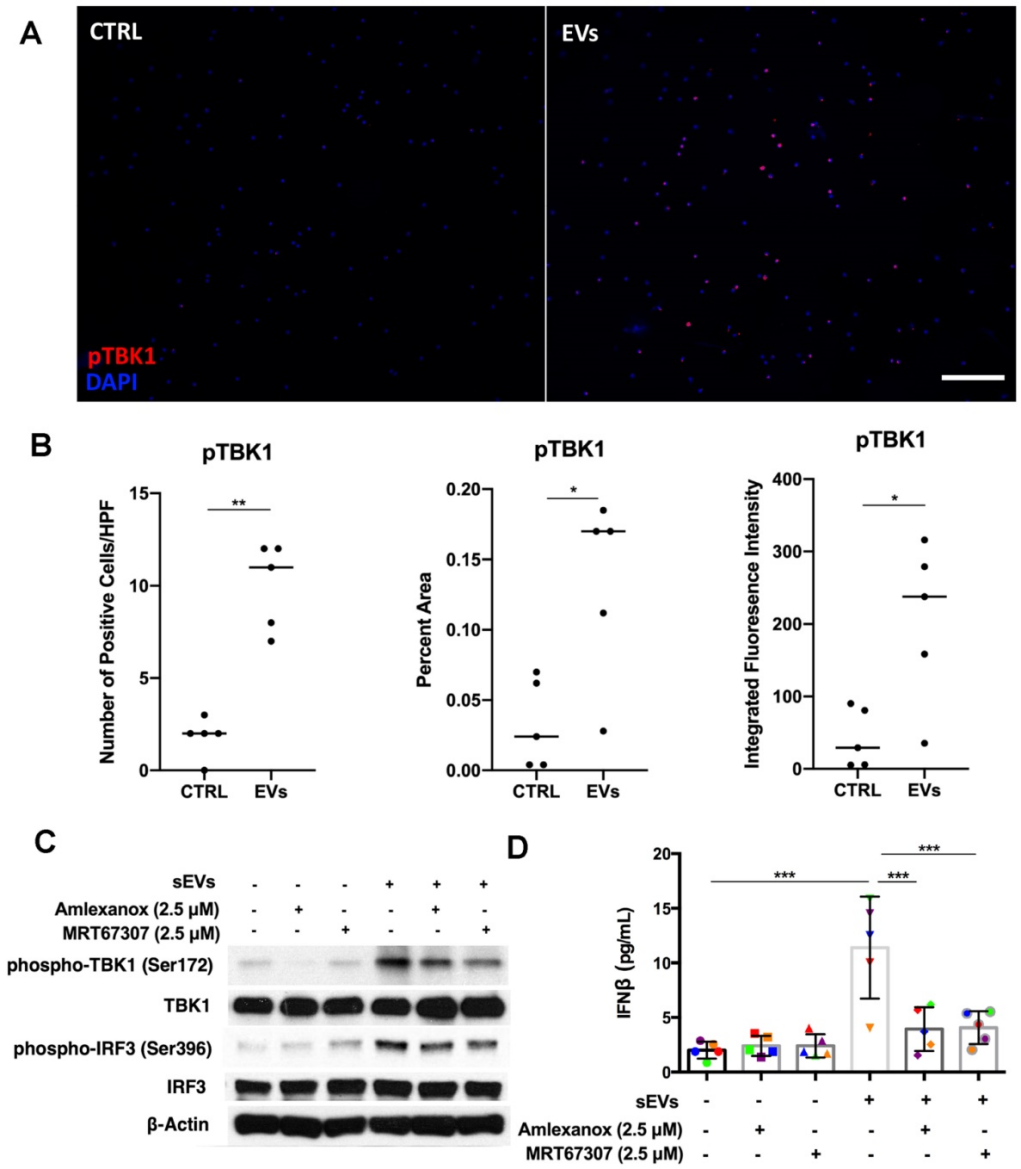
** Inhibition of TBK1 decreased DM plasma derived small extracellular vesicles' immunostimulatory effects in PBMCs.** (A) Representative immunofluorescent staining images showing that sEVs derived from DM plasma induced phosphorylation of TBK1 in PBMCs. (Scale bar 100 µm) (B) Bar graphic depicting the relative intensity of phosphorylated TBK1 immunofluorescent staining in PBMCs with/without DM plasma-derived sEVs stimulation (n = 5). (C) TBK1 inhibitors suppressed DM plasma-derived sEVs induced TBK1 and IRF3 phosphorylation in PBMCs. (D) DM plasma-derived sEVs induced IFNβ release in PBMCs (11.40 ± 4.669 pg/mL, n = 5) when compared with untreated PBMCs (2.000 ± 0.7674 pg/mL, n = 5). 2.5 µM of Amlexanox (TBK1 inhibitor) pretreatment impaired sEVs-triggered IFNβ release in PBMCs (3.933 ± 2.002 pg/mL, n = 5); 2.5 µM of MRT67307 (TBK1 inhibitor) pretreatment impaired DM sEVs-triggered IFNβ release in PBMCs (4.067 ± 1.511 pg/mL, n = 5). Data in B represent median. Data in D represent mean ± SD. *P < 0.05, **P < 0.01, ***P < 0.001 between groups as indicated. Comparison between two groups was analyzed by the Student* t* test. Comparison among three or more groups was performed using ANOVA, followed by Student-Newman-Keuls test.

Previous studies have shown that kinase TBK1 phosphorylated by STING could activate IRF3 in the cytosolic DNA signaling pathway, inducing type I IFN production 14. After showing that STING was involved in DM sEVs-triggered type I IFN response by PBMCs, we next wanted to understand whether TBK1 also played a role in the STING-mediated type 1 IFN response. By using immunofluorescent staining, we confirmed that the EVs-stimulated PBMCs had more phospho-TBK1 positive fluorescence (**Figure 6A-B**). When PBMCs were pretreated with the TBK1 inhibitors Amlexanox or MRT67307, both inhibitors suppressed DM patients' plasma-derived sEVs-induced IFNβ release in PBMCs via inhibiting TBK1 and its downstream protein IRF3 phosphorylation (**Figure 6C-D**). Similar results were also seen with TNFα (**Figure S10**).

Collectively, these results showed that as a downstream signaling protein of STING, TBK1 was also involved in the proinflammatory response that is triggered by DM patients' plasma-derived sEVs.

### Digestion of DM EVs-captured DNA impaired STING mediated proinflammatory cytokine release

After establishing the role of DM sEVs in the activation of the STING pathway and subsequent IFNβ production, we investigated the mechanism by which sEVs activated this pathway. As STING is activated by double stranded DNA (dsDNA), we hypothesized that sEVs may capture dsDNA which in turn stimulates the STING-mediated type I IFN response 26. DNA captured by EVs appears to exist not only on the surface, but also within the lumen 26. DNase treatment could not completely digest EVs-captured DNA unless the EVs were treated with DNase and lipid destabilizing agent (Triton X-100). As DNase I digests not only dsDNA, but also ssDNA, to further confirm the role of dsDNA captured by sEVs in STING-mediated type I IFN response, we pretreated sEVs with dsDNase instead of DNase I.

**Figure 7:**
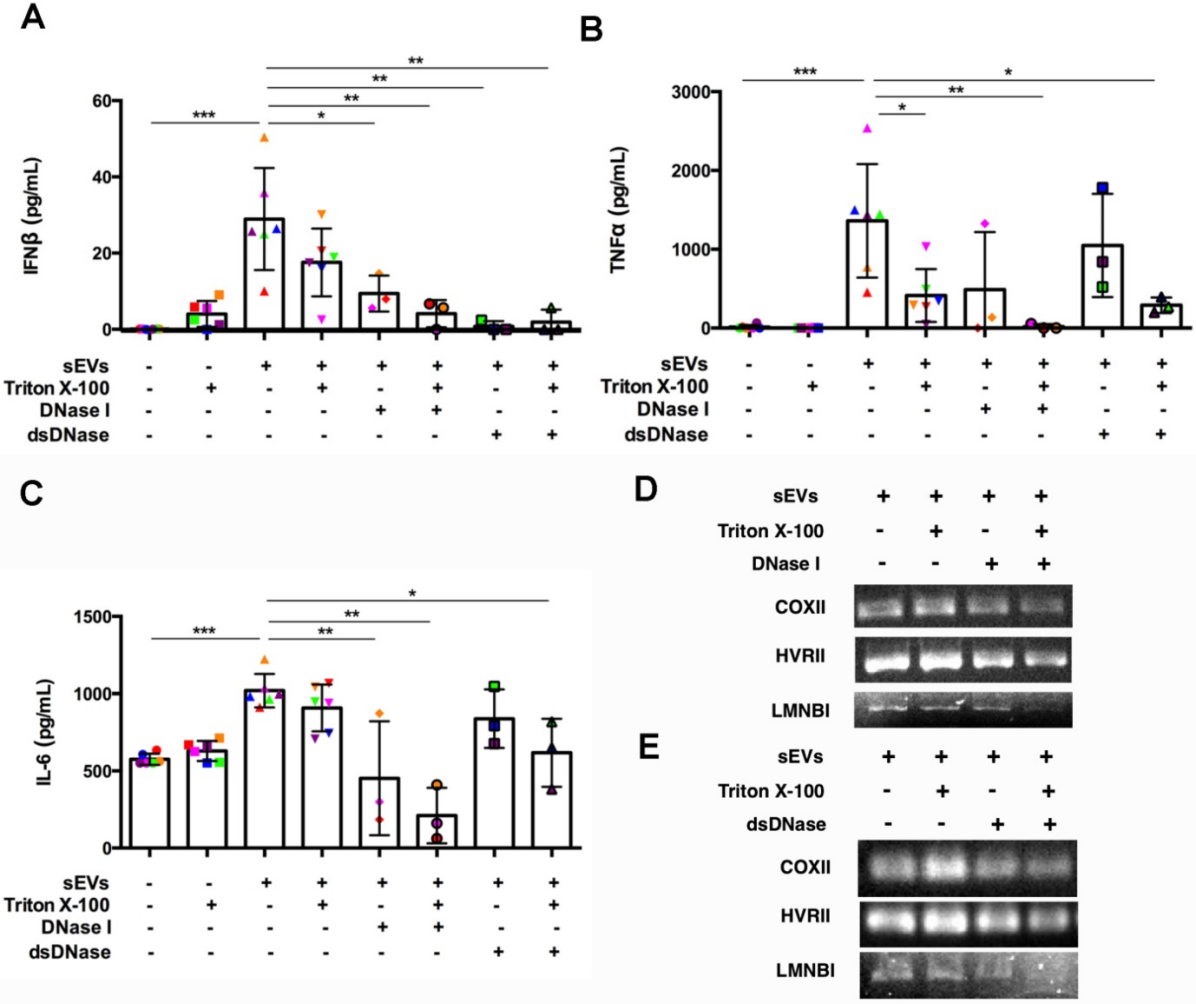
** Digestion of DM plasma-derived small extracellular vesicles-captured DNA impaired their triggered pro-inflammatory response in PBMCs.** (A) Pre-treatment with Triton X-100 and DNase (DNase I or dsDNase) attenuated sEVs ability to trigger IFNβ release in PBMCs (n = 3-6). (B) Pre-treatment with Triton X-100 and DNase (DNase I or dsDNase) attenuated sEVs ability to trigger TNFα release in PBMCs (n = 3-6). (C) Pre-treatment with Triton X-100 and DNase (DNase I or dsDNase) attenuated sEVs ability to trigger IL6 release in PBMCs (n = 3-6). (D) Genomic/mitochondrial DNA captured by EVs in the presence/absence of 0.075% Triton X-100 with/without DNase I was assessed by PCR using relative specified primers. (E) Genomic/mitochondrial DNA captured by EVs in the presence/absence of 0.075% Triton X-100 with/without dsDNase was assessed by PCR using relative specified primers. Data in (A,B,C) represent mean ± SD. *P < 0.05, **P < 0.01, ***P < 0.001 between groups as indicated. Comparison among three or more groups was performed using ANOVA, followed by Student-Newman-Keuls test.

**Figure 8:**
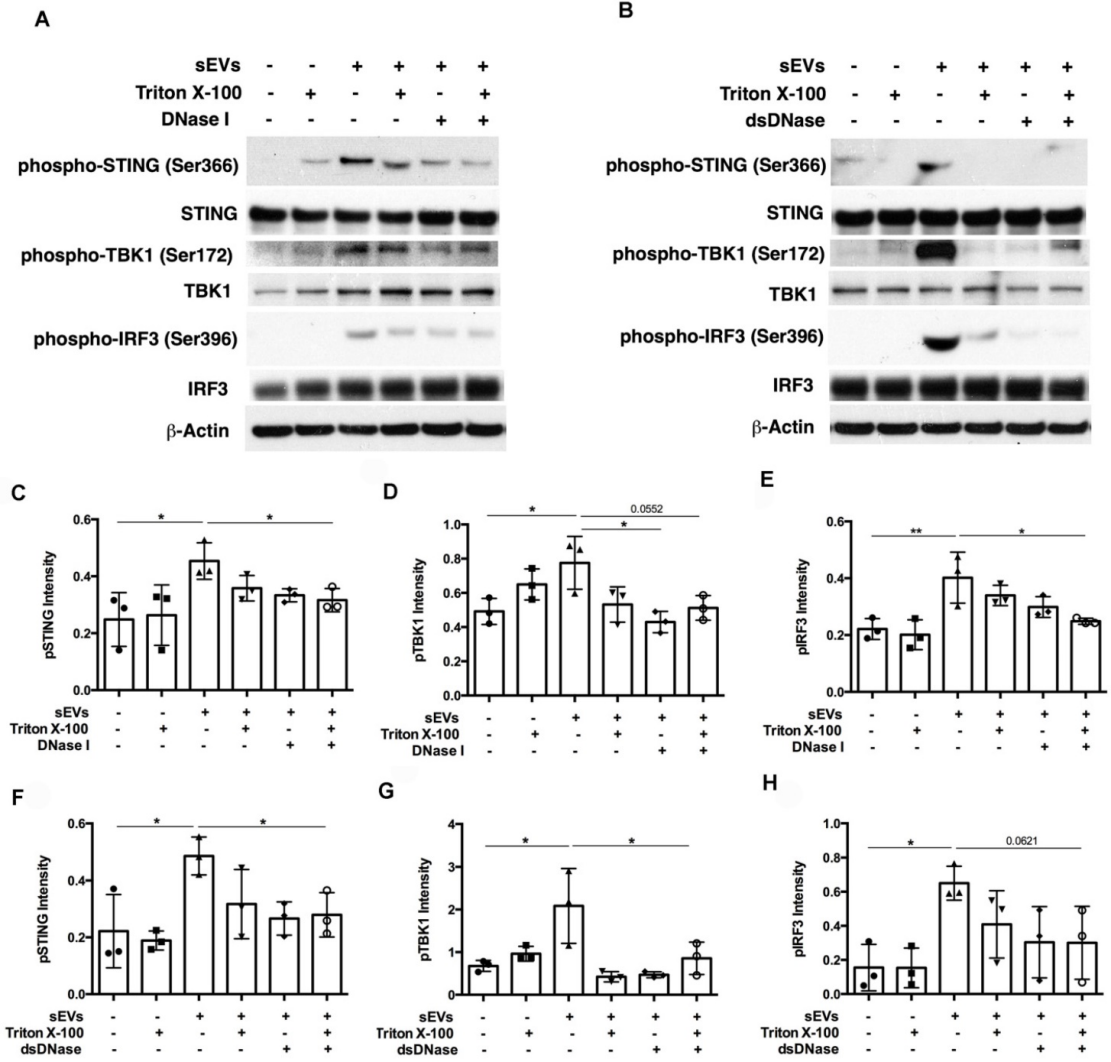
** Digestion of DM plasma-derived small extracellular vesicles-captured DNA impaired their triggered STING signaling pathway activation in PBMCs.** (A) The effects of DM plasma derived sEVs pretreated in the presence/absence of 0.075% Triton X-100 with/without DNase I on STING phosphorylation and its downstream signaling pathway TBK1, and IRF3 phosphorylation in PBMCs. (B) The effects of DM plasma derived sEVs pretreated in the presence/absence of 0.075% Triton X-100 with/without dsDNase on STING phosphorylation and its downstream signaling pathway TBK1, and IRF3 phosphorylation in PBMCs. Relative intensity of phosphorylated STING (C), phosphorylated TBK1(D), and phosphorylated IRF3 (E) in PBMCs stimulated by DM plasma derived sEVs pretreated in the presence/absence of 0.075% Triton X-100 with/without DNase I (n = 3). Relative intensity of phosphorylated STING (F), phosphorylated TBK1(G), and phosphorylated IRF3 (H) in PBMCs stimulated by DM plasma derived sEVs pretreated in the presence/absence of 0.075% Triton X-100 with/without dsDNase (n = 3). Data were represent mean ± SD. *P < 0.05,**P < 0.01 between groups as indicated. Comparison among three or more groups was performed using ANOVA, followed by Student-Newman-Keuls test.

SEVs pretreated by DNaseI and Triton X-100 or dsDNase and Triton X-100 attenuated DM plasma-derived sEVs-induced IFNβ, TNFα, and IL-6 release by PBMCs (**Figure 7A-C**). Polymerase chain reaction (PCR) was used to amplify indicated regions of the mitochondrial genome (*COXII* and *HVRII*) and nuclear genes (*LMNBI*) from sEVs. When compared with untreated sEVs, DNase I pretreatment reduced mitochondrial genes and nuclear genes levels. Moreover, sEVs pretreated with DNase I and Triton X-100 decreased mitochondrial gene and nuclear gene levels more than DNase I alone (**Figure 7D**). sEVs pretreated with dsDNase with/without Triton X-100 had similar results (**Figure 7E**). Moreover, sEVs pretreated with DNase I, Triton X-100, or DNase I and Triton X-100 attenuated STING phosphorylation and downstream signaling proteins TBK1 and IRF3 phosphorylation (**Figure 8A, 8C-E**). Immunoblot results further supported these results, with sEVs pretreated with dsDNase or Triton X-100 or dsDNase and Triton X-100 having similar effects to DNaseI (**Figure 8B, 8F-H**).

In summary, our results suggest that sEVs-captured dsDNA contributed to the STING-mediated proinflammatory response by PBMCs, since digestion of DNA captured by sEVs attenuated STING-mediated proinflammatory effects.

## Discussion

Increasing amounts of research suggests that the type I IFN signature likely contributes to DM pathogenesis 8, 36, 37. Patients with DM have an increased type I IFN signature in the muscle, blood and skin, which has also been described in patients with SLE, RA, Sjogren's syndrome and system sclerosis 5, 38. In addition, type I IFN-producing dendritic cells are abundant in DM tissues 39. When compared with other types of inflammatory myopathies, the overexpression of type I IFN inducible transcripts and proteins in muscle tissue is a unique feature of DM. The iatrogenic administration of recombinant IFNs has also been reported to induce DM 8. IFNs are critical determinants in immunocompetence and autoimmunity, capable of enhancing antigen presenting cells, differentiating lymphocytes, and are endogenously regulated by a low-level constitutive feedback loop capable of self-amplification 40. STING works as a critical sensor and adaptor for the host immune response to cytosolic DNA and cyclic dinucleotides in type I IFN signaling 41. Overactivation of the STING signaling pathway has recently been linked with many autoimmune diseases with similar features to DM such as type I interferonopathies including STING-associated vasculopathy with onset in infancy (SAVI) and lupus erythematous (LE) 42. However, few studies have examined the role of STING in DM pathogenesis 43, 44. Whether and how the STING signaling pathway may affect the systemic type I IFN response in DM remains elusive.

Our findings characterized the innate immune pathways and sensors/adaptors that increase type I IFN signaling and are key to instigating autoinflammatory events in DM immune cells. We found that circulating EVs may play a critical role in triggering a STING-mediated type I IFN response in DM immune cells. EVs are produced by a multitude of cells and mediate the inflammatory response via presentation of antigens and activation of various toll like receptors 38. They have been linked to autoimmune conditions such as RA, Sjoren's syndrome, Systemic Sclerosis, and LE, with the belief that they serve as sources of self-antigens and peptide-MHC complexes capable of promoting autoantibodies production and autoreactive T cells activation 38. We have also found a role for EVs in DM, as inducers of STING, type I IFNs, and proinflammatory cytokines in PBMCs. Our findings that EVs can activate the STING signaling pathway are consistent with Deschamps et al., who reported that EVs released by herpes simplex virus 1-infected cells blocked virus replication in recipient cells in a STING-dependent manner 45. Similarly, in LE, Kato et al., recently reported apoptosis derived vesicles can enhance type I IFN production in a STING-dependent manner suggesting the importance of vesicle mediated STING activation in autoimmunity 42.

In the current study, we also found that DM plasma contains increased numbers of smaller, more complex (based on surface marker expression) EVs than HC plasma. Size differences in EVs may be of importance as it may have implications on biologic function. Exosomes (30 nm-150 nm) are likely important for antigen presentation while microvesicles (100 nm-1000 nm) may function predominantly in cell-cell communication 46. The increased, smaller EVs in DM consistent with exosomes could therefore directly increase antigen presentation and immune stimulation. Our findings may also have important implications for DM clinical management. The number of plasma sEVs was elevated in DM patients compared to HC, and also increased with disease severity; sEVs, but not lEVs, correlated with CDASI activities, a reliable, validated outcome measure to quantify disease severity in DM patients. Further studies are necessary to examine the relationships, including temporal associations, between the number of EVs and disease severity, as an increased number of EVs may be a precursor to a DM flare and serve to guide treatment options. Content analysis of EVs may be useful, as proteins captured by EVs can serve as biomarkers for DM and disease complications 47, 48.

The EVs derived from DM plasma triggered a pro-inflammatory response with STING phosphorylation. Selective inhibition of the STING signaling pathway significantly attenuated DM patients' plasma-derived EVs-mediated pro-inflammatory effects. These stimulatory results provide evidence for a role of EVs in promoting STING dependent type I IFN and cytokine production. The ability of the STING antagonists to modulate production of both type I IFN and other cytokines may be of therapeutic use, as these cytokines are associated with DM pathogenesis. Research regarding conceptual STING targeted therapy has already been tested in mice, with positive preliminary results eliminating disease in Fcgr2b-deficient lupus mice 49. Effects on pro-inflammatory cytokines in addition to the type I IFN response may be explained by the diverse IRF3 independent downstream effects of STING; TBK1 may do more than just phosphorylate IRF3, it could also recruit STAT6 to the ER for subsequent phosphorylation by TBK1, leading to induction of STAT6-dependent anti-viral genes 50. It is also feasible that STING directs TBK1 to phosphorylate and activate NFκB in response to cytosolic DNA 14. TBK1 associated with STING is also shown to participate in dsDNA-mediated canonical activation of NF-κB, similar to IRF3, to promote gene transcription of proinflammatory cytokines in a TRAF6 dependent manner. A novel dsDNA-mediated NF-κB activation pathway facilitated through a STING-TRAF6-TBK1 axis suggests a target for therapeutic interventions to avert dsDNA-mediated cytokine and type I IFN autoimmune disease 15. To further support the activation of STING and downstream effects, we confirmed that inhibition of TBK1, a protein downstream of STING, also impaired DM plasma-derived EVs triggered pro-inflammatory effect on circulating immune cells.

Our study delineated that DNA captured by circulating EVs contribute to STING-mediated proinflammatory effects in DM patients. The mechanisms by which EVs stimulate STING may relate to trafficking of dsDNA. Studies have shown EVs contain both genomic and mitochondrial DNA 51. The genomic material inside or attached to the membrane of these EVs may directly stimulate cytosolic cGAS within interacting immune cells 26. Recent studies have shown priming of dendritic cells by T cells, mediated through STING activation of EVs that contain genomic and mitochondrial DNA 26. During states of acute or chronic inflammation, cellular stress may promote EVs secretion and vascular permeability, and may enhance extracellular genomic and mitochondrial migration of resident inflammatory cells to initiate/propagate pathological IFN and cytokine production 52. A recent study also showed that mitochondrial stress or dysfunction could trigger mitochondrial DNA release into the cytosol, with activation of the cGAS-STING-mediated innate immune response. Mitochondria-associated vaccinia virus-related kinase 2 was essential for mitochondrial DNA-mediated innate immune response 53. Besides, potent activators of the STING pathway may also include self-DNA that has leaked from the nucleus of the host cell, perhaps following cell division or as a consequence of DNA damage due to cellular stress 10. This self-DNA may be in the form of NETs, as they were found to be enriched in oxidized mitochondrial DNA, which could stimulate the production of type I IFN through the cGAS/STING signaling pathway and contribute to lupus-like disease 54. Furthermore, it was observed that LL-37 could protect NET-derived DNA in SLE, and could help transport extracellular self-DNA into monocytes to stimulate the cGAS/STING pathway 55, 56. A recent study also showed that the cytosolic DNA sensor cGAS could recognize NETs and mediate immune cell activation during infection 57. We believe further studies will be required to help understand the role of circulating EVs mediated type I IFN in DM pathogenesis.

In conclusion, our current study found that EVs derived from DM patients' plasma triggered proinflammatory response with STING phosphorylation. Suppression of STING signaling pathway significantly attenuated DM plasma-derived EVs-mediated proinflammatory effects. Targeting STING might provide insight into a potential therapeutic approach for DM.

## Supplementary Material

Supplementary figures and tables.

## Abbreviations

CDASI: cutaneous dermatomyositis disease area and severity index
DAPI: 4',6-diamidino-2-phenylindole
DM: dermatomyositis
dsDNA: double stranded DNA
ELISA: enzyme-linked immunosorbent assay
ER: endoplasmic reticulum
FBS: fetal bovine serum
HC: healthy controls
IFN: interferons
ILD: interstitial lung disease
IRF3: interferon regulatory factor 3
PBMCs: peripheral blood mononuclear cells
PFA: paraformaldehyde
PVDF: polyvinylidene fluoride
RA: rheumatoid arthritis
RIPA: radioimmunoprecipitation assay buffer
RLRs: RIG-I-like receptors
SAVI: STING-associated vasculopathy with onset in infancy
SD: standard deviations
SDS-PAGE: sodium dodecyl sulfate-polyacrylamide gel electrophoresis
SLE: systemic lupus erythematosus
STING: stimulator of interferon genes
TBK1: TANK-binding kinase-1
TGB: thioglycolate broth
TLRs: toll-like receptors
