# Supplementary material for: Plasma-derived DNA containing-extracellular vesicles induce STING-mediated proinflammatory responses in dermatomyositis
Source: Theranostics. 2021 May 21;11(15):7144–58. doi: 10.7150/thno.59152 (PMC8210592; doi:10.7150/thno.59152)
Supplement: Supplementary file 1 — Supplementary figures and tables. [file thnov11p7144s1.pdf]

Supplementary Material

Plasma-derived DNA containing-extracellular vesicles induce STING-mediated proinflammatory responses in dermatomyositis

Yubin Li[1,2], Christina Bax[1,2], Jay Patel[1,2], Thomas Vazquez[1,2], Adarsh Ravishankar[1,2], Muhammad M. Bashir[1,2], Madison Grinnell[1,2], DeAnna Diaz[1,2], Victoria P. Werth[1,2,*]

[1]Corporal Michael J. Crescenz Veterans Affairs Medical Center Philadelphia, PA

[2]Department of Dermatology, School of Medicine, University of Pennsylvania, Philadelphia, PA 19104

[*]**Corresponding Author:**

Victoria P. Werth, M.D.

Department of Dermatology, University of Pennsylvania,

Perelman Center for Advanced Medicine, Suite 1-330A,

3400 Civic Center Boulevard, Philadelphia, PA 19104, USA.

Tel: +1 215 823 4208, Fax: +1 866 755 0625

E-mail: werth@pennmedicine.upenn.edu

Keywords

Extracellular vesicles; dsDNA; type I interferon; Stimulator of interferon genes; Dermatomyositis

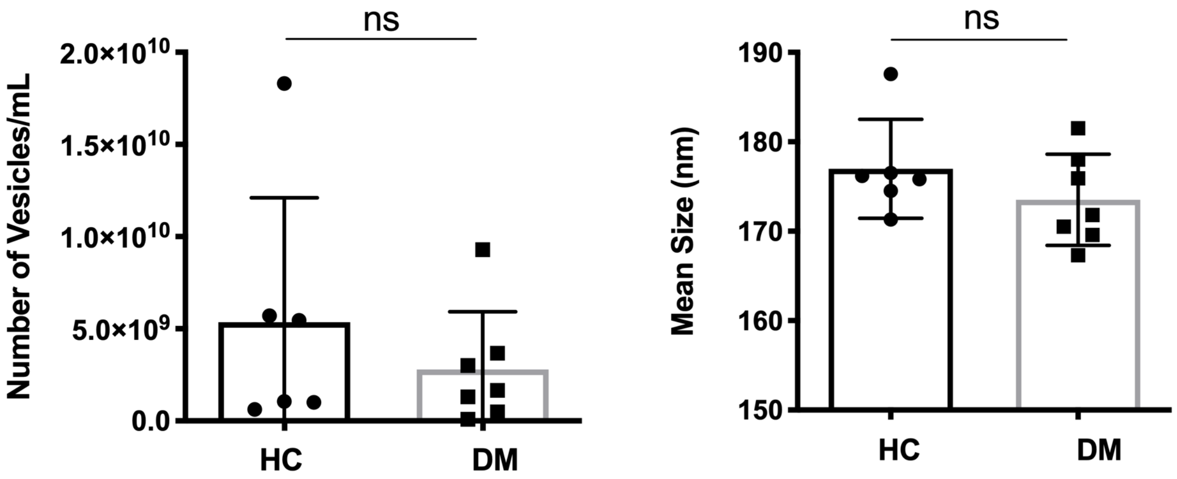

Figure S1. The concentration and average size of large extracellular vesicles-derived from DM plasma and HC plasma.

(A) Total number of lEVs derived from healthy plasma ($5.358 \times 10^9 \pm 6.745 \times 10^9$ particles/mL, n = 6) and DM plasma ($2.786 \times 10^9 \pm 3.140 \times 10^9$ particles/mL, n = 7). (B) Average size of lEVs derived from healthy plasma (177.0 ± 5.539nm, n = 6) and DM plasma (173.5 ± 5.092 nm, n = 7). Data were represented as mean ± SD. Comparison between two groups was analyzed by the Student *t* test.

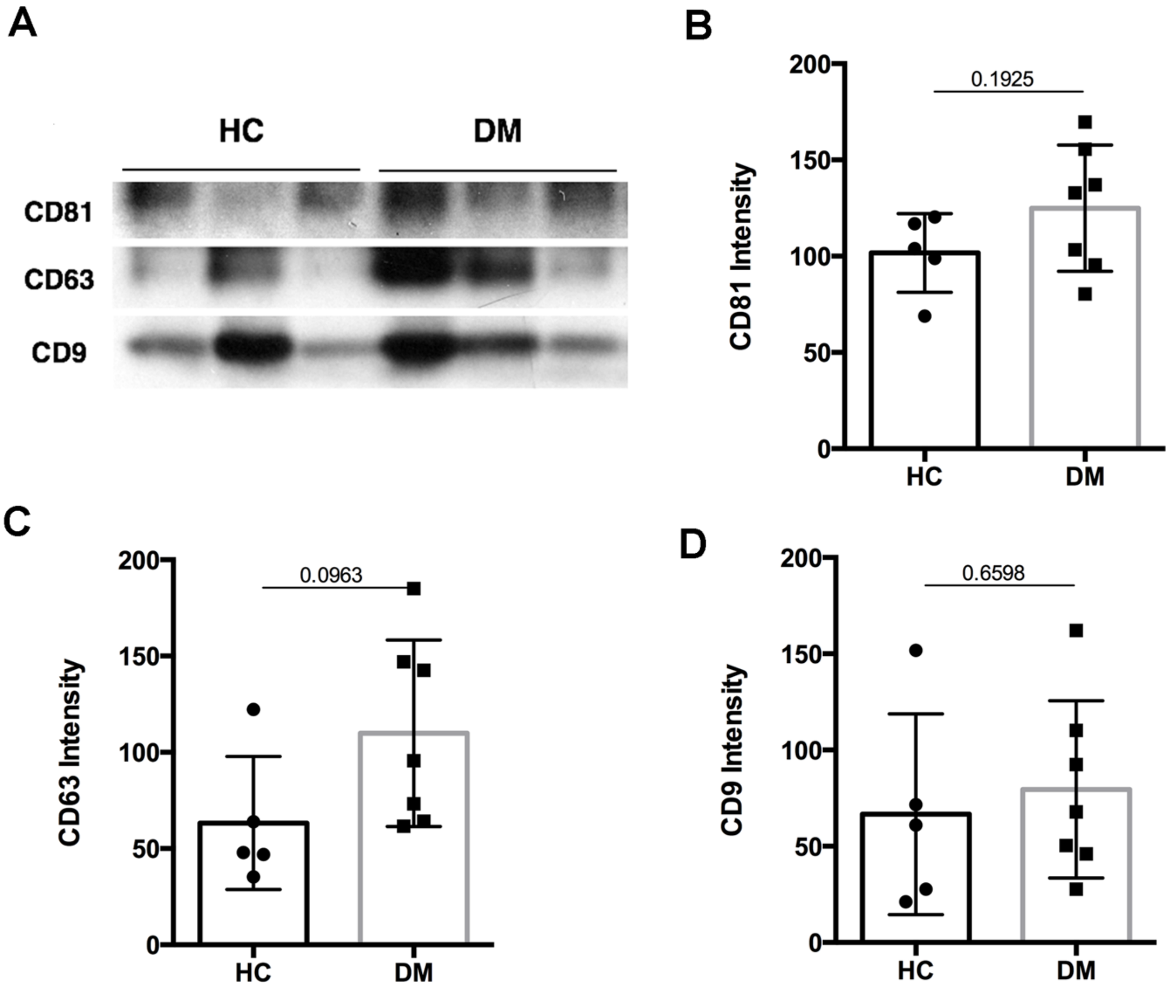

Figure S2. Same number of DM plasma and HC-derived small extracellular vesicles exhibited similar levels of small extracellular vesicles surface markers.

(A) Immunoblot image showed different surface makers CD81, CD9, CD63 expression level from same number ($3.125x10^8$ particles) of sEVs derived from HC plasma (n = 3) and DM plasma (n = 3). (B) Relative intensity of CD81 expression level from same number of sEVs derived from HC plasma (n = 5) and DM plasma (n = 7). (C) Relative intensity of CD63 expression level from same number of sEVs derived from HC plasma (n = 5) and DM plasma (n = 7). (D) Relative intensity of CD9 expression level from same number of sEVs derived from HC plasma (n = 5) and DM plasma (n = 7). Data were represented as mean ± SD. Comparison between two groups was analyzed by the Student *t* test.

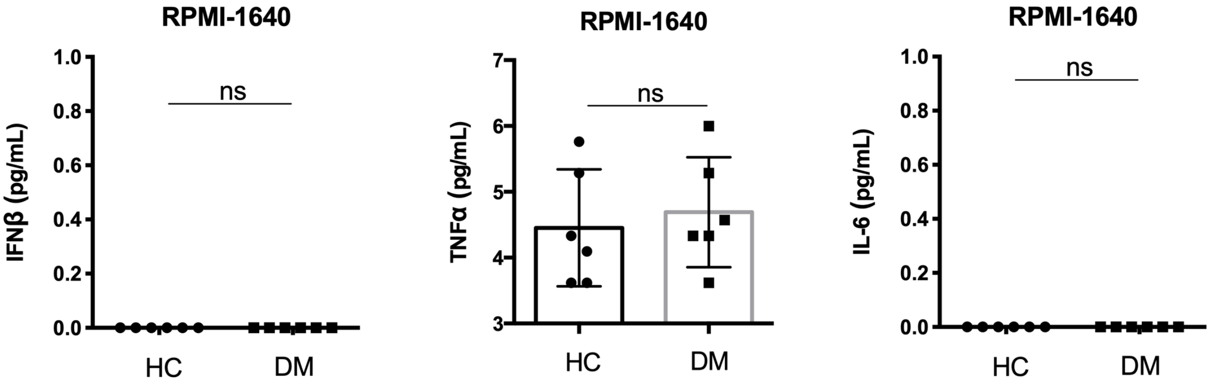

Figure S3. Proinflammatory cytokines captured by HC or DM plasma-derived EVs. Both HC and DM plasma-derived EVs captured IFNβ and IL-6 were undetectable, TNFα captured by HC and DM plasma-derived EVs is very low and has no significant difference. HC plasma-derived EVs ($n = 6$) and DM plasma-derived EVs ($n = 6$). Data were represented as mean ± SD. Comparison between two groups was analyzed by the Student *t* test.

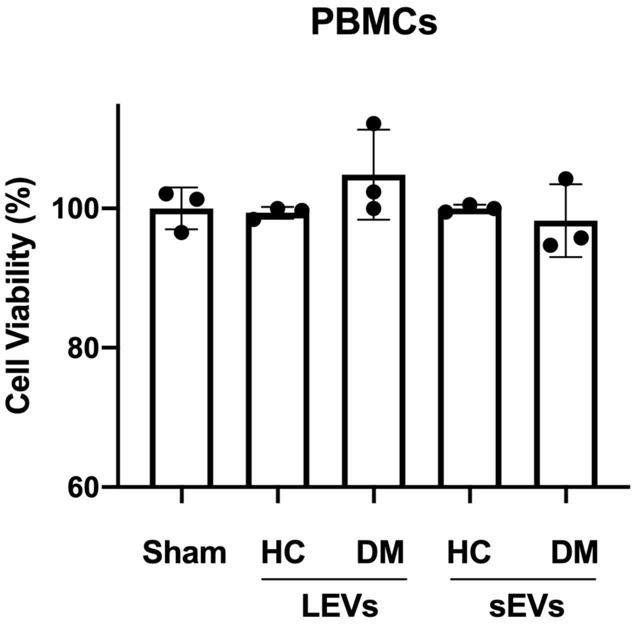

Figure S4. HC or DM plasma-derived EVs had no effects on cell viability of PBMCs. PBMCs were placed in 96-well plates at a density of 1.5×10^6 cells/mL, 100 µL/well. After stimulation of cells with 5 µL of large EVs or small EVs derived from 50 µL of HC or DM plasma for 15 h, the plates were incubated with 10 µL/well of WST-8 in the incubator with 95% air and 5% CO_2 at 37°C for 1 h, and then measured at 460 nm absorbance wavelength. HC plasma-derived EVs (n = 3) and DM plasma-derived EVs (n = 3) were studied. Data are represented as mean ± SD. Comparison between two groups was analyzed by the Student *t* test.

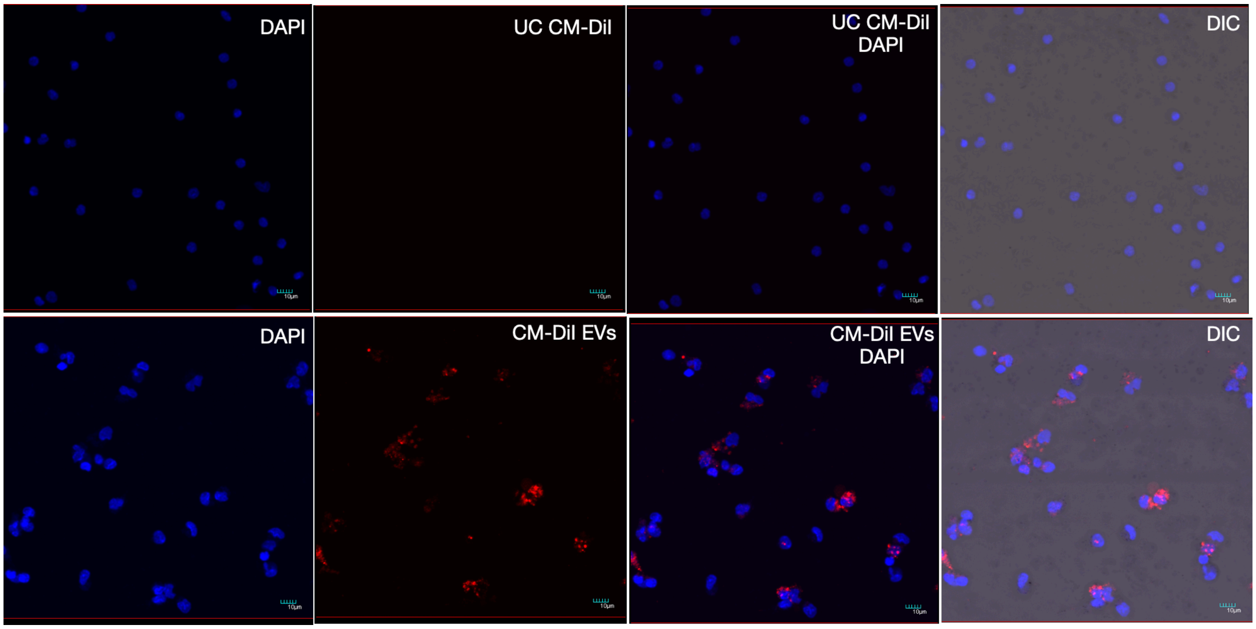

Figure S5. EVs internalized by PBMCs.
Cells were stimulated with 12.5 µL of precipitated CM-DiI dye following ultracentrifugation or CM-DiI dye-labeled EVs for 15 h. The cells were fixed, incubated with 1 µg/mL of DAPI in PBS for 1 min, washed 3 times, and then studied by using confocal microscopy. PBMCs were placed in EZ slide at a density of $1.5x10^6$ cells/mL, 500 µL/well. The upper panel showed no interaction of the precipitated CM-DiI dye with PBMCs; the lower panel showed that CM-DiI dye-labeled EVs could be properly internalized by PBMCs (Scale bar = 10 µm).

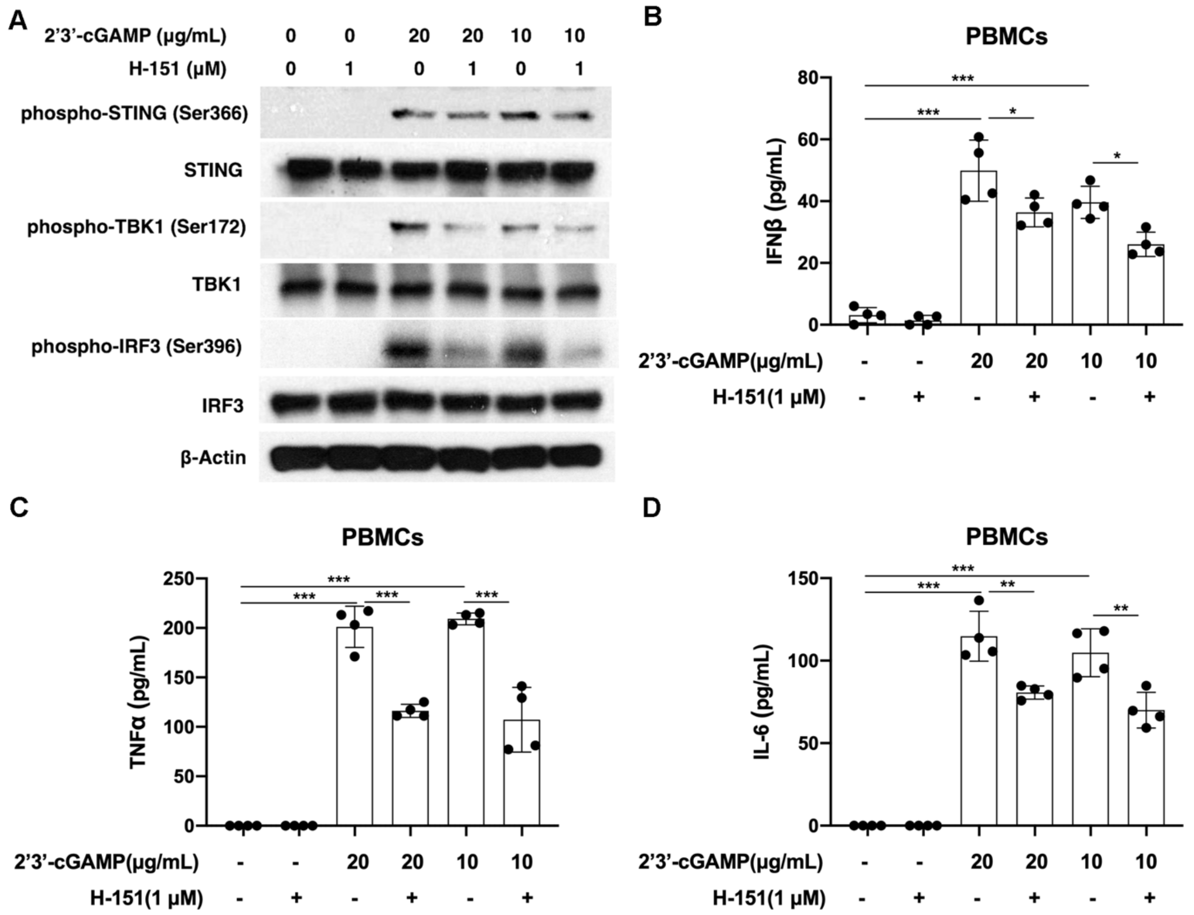

Figure S6. H-151 suppressed 2’3’-cGAMP-induced STING signaling pathway phosphorylation and proinflammatory cytokine release in PBMCs.

PBMCs were placed in 6-well plates at a density of 1.5×10^6 cells/mL, 2 mL/well. The cells were pretreated with/without 1 µM of H-151 for 1 h, and then stimulated in the presence/absence of 10 µg/mL or 20 µg/mL of 2’3’-cGAMP for 15 h. After stimulation, the cells were collected for immuno-blot to check the changes in the STING signaling pathway. ELISAs were performed to examine proinflammatory cytokines release in the supernatants. Data in (B,C,D) represent mean ± SD. **P* < 0.05 ***P* < 0.01 ****P* < 0.001 between groups as indicated. Comparison among three or more groups was performed using ANOVA, followed by Student-Newman-Keuls test.

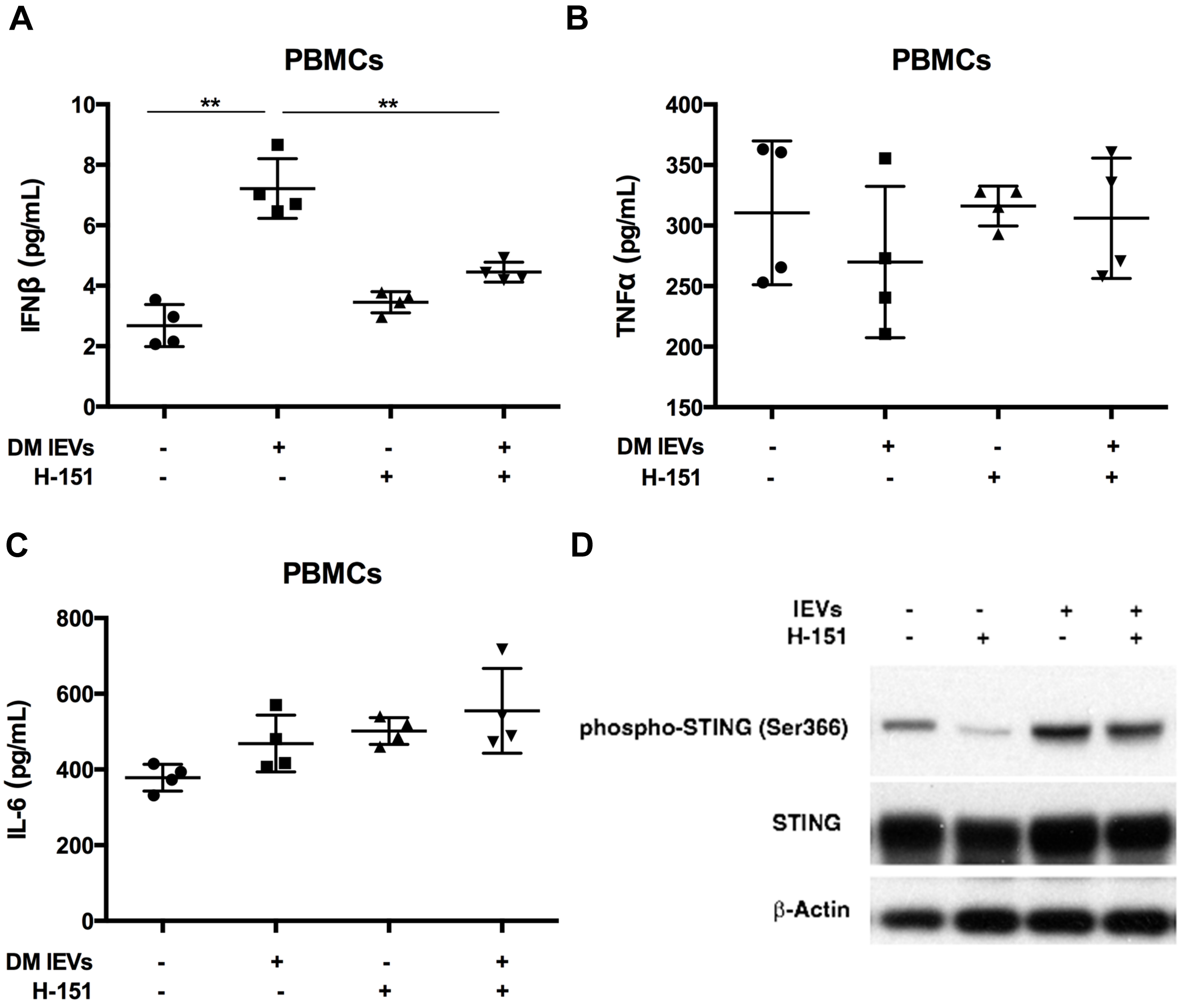

Figure S7. DM plasma-derived large extracellular vesicles induced STING-mediated IFNβ production in PBMCs.

(A) lEVs derived from DM plasma induced IFNβ release in PBMCs. H-151 attenuated lEVs-triggered IFNβ release in PBMCs. (B) STING antagonist H-151 and lEVs derived from DM plasma had no effect on TNFα or (C) IL6 release in PBMCs. (D) Immunoblot image showed that lEVs derived from DM plasma induced Serine 366 phosphorylation of STING in PBMCs. STING antagonist H-151 attenuated lEVs-triggered STING phosphorylation. Data in (A,B,C) represent mean ± SD. $^{**}P < 0.01$ between groups as indicated. Comparison among three or more groups was performed using ANOVA, followed by Student-Newman-Keuls test.

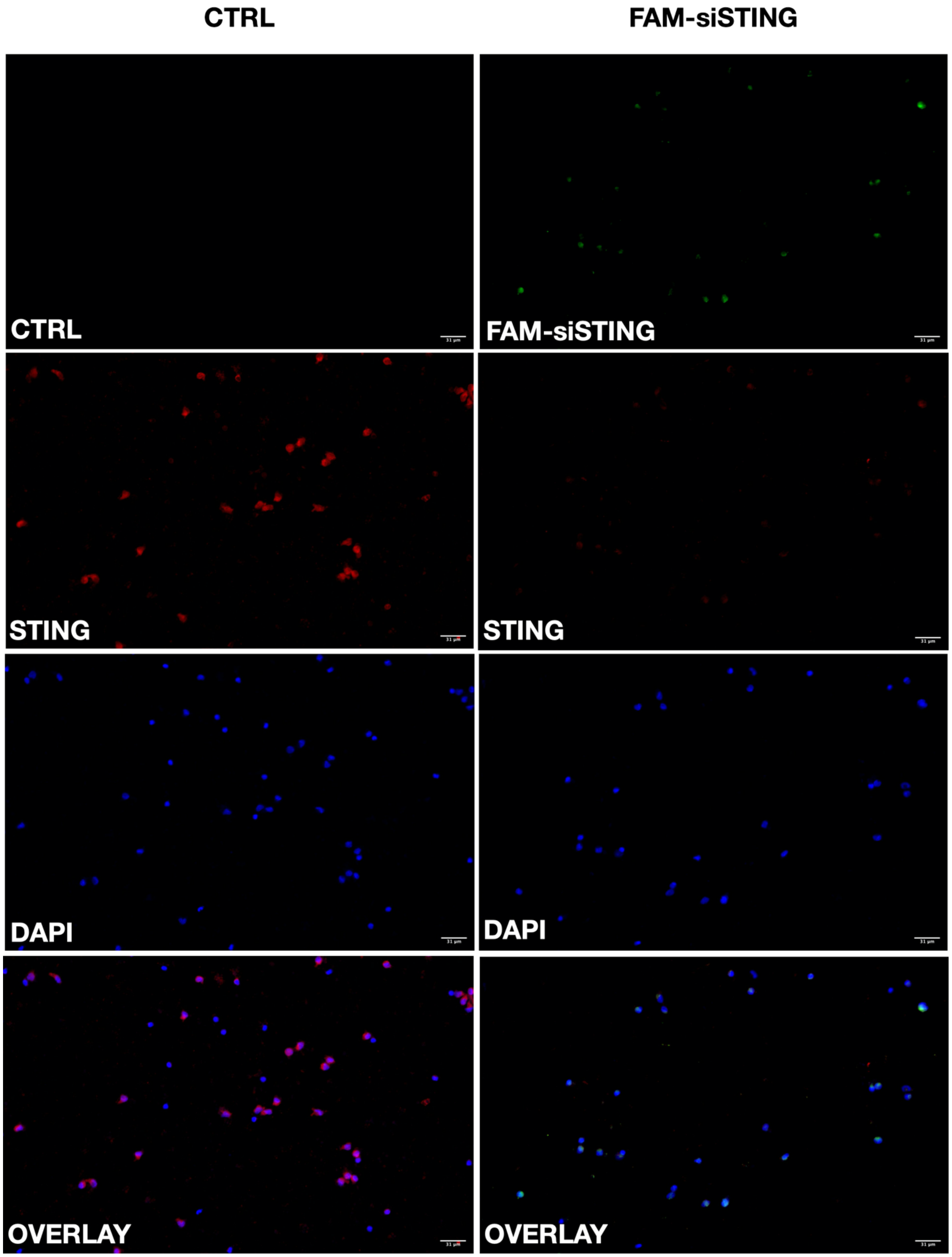
CTRL
FAM-siSTING
CTRL
FAM-siSTING
STING
STING
DAPI
DAPI
OVERLAY
OVERLAY

Figure S8. FAM-siSTING could be transfected and decreased STING expression in PBMCs.
PBMCs were placed in EZ slide at a density of $1.5x10^6$ cells/mL, 500 μL/well. After cells were transfected by FAM-labelled siSTING for 36 h, the cells were fixed, permeabilized, and stained for STING. Cells were then incubated with 1 μg/mL of DAPI in PBS for 30 s, washed 3 times, and then studied by using fluorescent microscopy. (Scale bar = 31 μm).

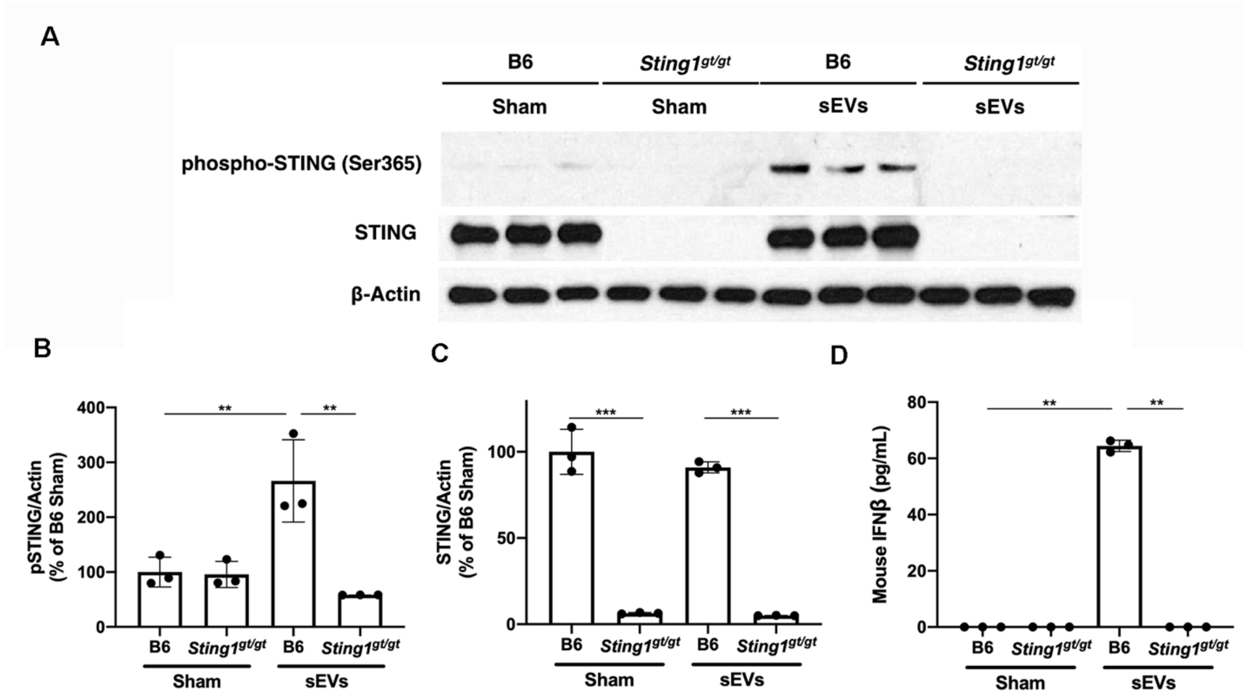

Figure S9. DM plasma-derived sEVs triggered STING phosphorylation with IFNβ production in WT (B6) mice eluted peritoneal macrophages other than STING KO (*Sting1$^{gt/gt}$*) mice peritoneal macrophages.

WT mice and STING KO mice eluted peritoneal macrophages were stimulated with/without 50 μL of DM plasma-derived sEVs for 15 h. (A) Immunoblot image showed that DM plasma-derived sEVs triggered STING phosphorylation in B6 mice eluted peritoneal macrophages other than *Sting1$^{gt/gt}$* mice eluted macrophages (B). B6 mice expressed total STING while *Sting1$^{gt/gt}$* mice dose not expressed total STING (C). sEVs triggered more IFNβ production in B6 macrophages but not in *Sting1$^{gt/gt}$* macrophages (D). Data in (B,C,D) represent mean ± SD. $^{**}P$ < 0.01 $^{***}P$ < 0.001 between groups as indicated. Comparison among three or more groups was performed using ANOVA, followed by Student-Newman-Keuls test.

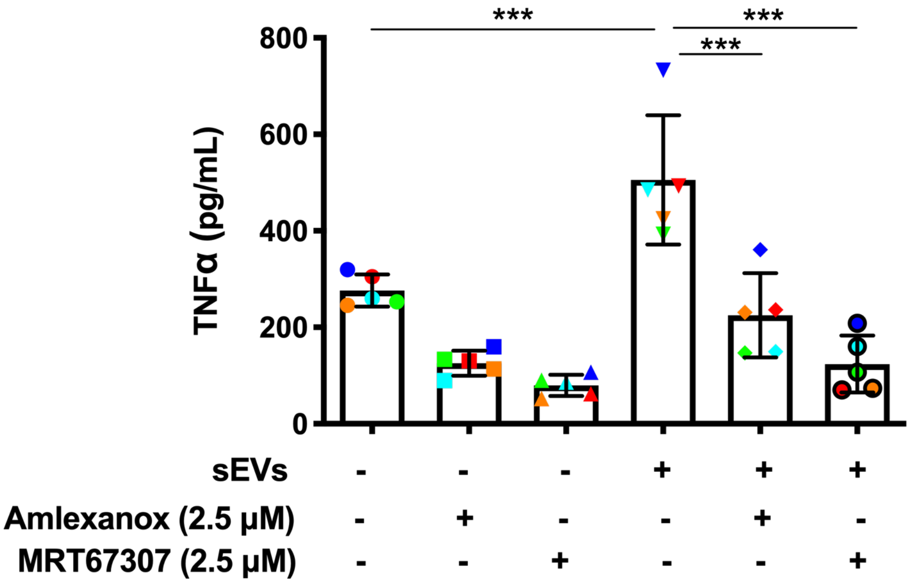

Figure S10. TBK1 inhibitors impaired DM plasma-derived sEVs triggered TNFα release in PBMCs.

DM plasma-derived sEVs induced TNFα release in PBMCs (505.7 ± 59.81 pg/mL, n = 5) when compared with untreated PBMCs (276.5 ± 14.92 pg/mL, n = 5). 2.5 µM of Amlexanox (TBK1 inhibitor) pretreatment impaired sEVs-triggered TNFα release in PBMCs (225.2 ± 39.04 pg/mL, n = 5); 2.5 µM of MRT67307 (TBK1 inhibitor) pretreatment impaired DM sEVs-triggered TNFα release in PBMCs (124.0 ± 26.51 pg/mL, n = 5). Data was represented as mean ± SD. ****$P < 0.001$ between groups as indicated. Comparison among three or more groups was performed using ANOVA, followed by Student-Newman-Keuls test.

Table S1. Patient characteristics

Patient number	Age	Sex	sEVs concentration ($X10^{11}$/mL plasma)	CDASI-A	Systemic therapy	Topical Therapy
**1**	72	F	12.6	9	MMF	CS
**2**	46	F	6.1	10	HCQ	CS
**3**	75	F	4.55	0		CS
**4**	19	M	11.8	11	Pred, HCQ, MMF, IVIG	CS, CI
**5**	18	M	15.5	24	Pred	
**6**	74	M	16.8	6		CI
**7**	62	F	15.7	35	Pred	CS, CI
**8**	63	F	20.6	27		CS, CI
**9**	60	F	9.97	5	MTX	CS
**10**	45	F	17.7	15	HCQ	CS
**11**	27	F	4.8	9	Pred	CI

Abbreviations: sEVs, small extracellular vesicles; CDASI-A, Cutaneous Dermatomyositis Disease Area and Severity Index Activity Score; MMF, mycophenolate mofetil; HCQ, hydroxychloroquine; Pred, prednisone; MTX, methotrexate; CS, corticosteroid; CI, calcineurin inhibitor.
